# Sequence-Based Models for RNA–Protein Interactions Imputation Might Be Insufficient for Novel Signal Prediction in eCLIP Data

**DOI:** 10.3390/ijms27031192

**Published:** 2026-01-24

**Authors:** Arsenii K. Rybakov, Daniil A. Khlebnikov, Daria Y. Ovchinnikova, Arina I. Nikolskaya, Arsenii O. Zinkevich, Andrey A. Mironov

**Affiliations:** 1 Faculty of Bioengineering and Bioinformatics, Lomonosov Moscow State University, 1-73 Leninskie Gory, Moscow 119991, Russia; 2 Vavilov Institute of General Genetics of RAS, Gubkina Str. 3, Moscow 119333, Russia

**Keywords:** RNA–protein interactions, machine learning, sequence-based models, eCLIP, RNAcompete

## Abstract

Predicting specific RNA–protein interactions remains a challenging task. Despite the existence of numerous methods, a unified approach has yet to emerge. Additional difficulties emerge from the properties of in vivo IP experiments and their systematic biases, such as the overrepresentation of highly expressed RNAs. Here, we present the PLERIO machine learning framework, which utilizes eCLIP data for a single protein to reconstruct the full spectrum of its potential interactions with the cellular transcriptome (i.e., both highly expressed and lowly expressed RNAs). In an effort to extrapolate our methodology to a multi-protein paradigm for de novo prediction of RNA–protein interactions on proteins lacking available eCLIP data, we extended our approach to 220 cellular proteins. We then demonstrate that this approach might not be well tailored to the limitations of current in vivo immunoprecipitation data, and may only be meaningful for in vitro experiments such as RNAcompete.

## 1. Introduction

Interactions between RNA and proteins play a pivotal role in post-transcriptional regulation, alternative splicing, RNA stability, and its intracellular localization [1,2,3]. It has been observed that certain functions of non-coding RNAs may be facilitated by one or more nuclear proteins or their complexes [4,5,6]. Notably, this has been documented for XIST [7], NEAT, MALAT1, and HOTAIR [8]. Conversely, the absence or presence of protein interaction with non-coding RNA (CHD4 [5], PRC2 [6]) has been demonstrated to regulate the functions of certain proteins. Furthermore, proteins have been demonstrated to facilitate RNA interactions with chromatin [9]. Therefore, the problem of RNA–protein interactions requires careful study to grasp the systems biology of regulatory interactions in the cell.

At present, there are limited data on in vivo RNA–protein contacts. For instance, eCLIP [10] results are available for 120 proteins in the K562 cell line [11], with each dataset dominated by information on highly expressed RNAs. However, RNAs with low expression are of greatest interest for studying regulatory interactions.

**Figure 1:**
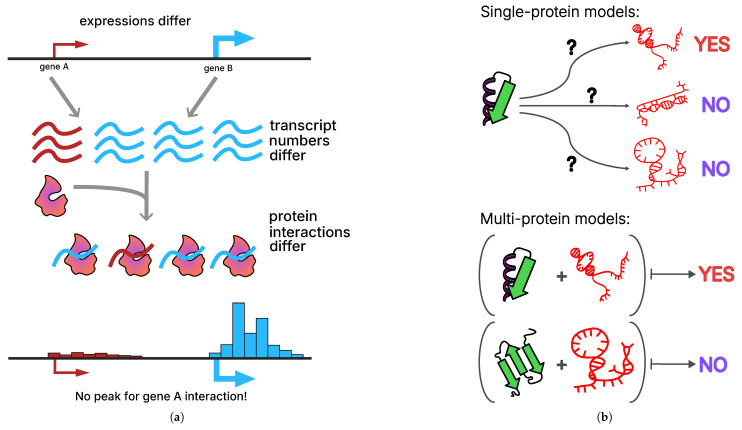
Conceptual challenges in predicting RNA–protein interactions from in vivo data. (**a**) Differences in gene expression lead to unequal transcript abundances, which in turn bias the observed frequency of RNA–protein interactions. High gene expression (gene B, blue) increases the likelihood of detecting RNA–protein interactions, whereas lowly expressed genes (gene A, red) may show few or no detectable interactions even if binding occurs. This results in the apparent absence of interaction peaks for lowly expressed genes. (**b**) Two conceptual modeling paradigms for RNA–protein interaction prediction: single-protein models evaluate binding between one protein and multiple RNA targets; multi-protein models consider multiple proteins and RNAs simultaneously, enabling the learning of shared binding patterns, knowledge transfer across proteins, and imputation of missing interactions.

Cross-linking immunoprecipitation (CLIP) methods facilitate the mapping of RNA-binding proteins (RBPs) at the transcriptome level in vivo. The immunoprecipitation protocol has long been known to exhibit certain flaws and artifacts [4]. For instance, there is a tendency for highly expressed RNAs to be over-represented in experimental data compared to controls. This sampling bias is introduced at the protocol level [12,13]. For reasons unrelated to the specificity of their interaction, highly expressed RNAs clog up more interactions of the assay target protein. Standard data processing protocols which permit the introduction of control background data into the pipeline serve to further exacerbate this issue. Even in scenarios where a portion of the experiment’s coverage originates from interactions between lowly expressed RNA and the target protein, there is no way to ascertain whether this coverage will surpass the statistical significance threshold necessary for determining the region of this RNA as an interaction peak [14,15] (Figure 1a). Additionally, eCLIP data is unable to differentiate between facultative interactions of a protein with RNA regions that are exclusively preferred in cooperative binding due to the single-protein nature of the elementary step of the protocol [16,17].

Furthermore, immunoprecipitation experiment data are also used in the context of predicting RNA–protein interactions de novo [18]. This task is commonly presented in two distinct classes. The first encompasses machine learning models that have been trained on RNA–protein interaction data for a specific protein. These models predict the probability of RNA–protein contact for a pair consisting of a protein and a novel RNA that the model has not previously encountered (see Figure 1b, top). The objective of these models is to identify characteristic sequence or structural motifs preferred by a given protein. However, the “one protein–one model” approach is inherently limited in its ability to generalize the patterns of RNA–protein interactions for proteins that were not included in the training data. The utility of these models is primarily limited to supplementing the existing interactome with various semi-extractable or weakly expressed transcripts. Consequently, the utilization of these models in identifying de novo interactions is constrained by the principles of homology.

To overcome these limitations, attempts can be made to create multi-protein models that are trained on multiple eCLIP datasets simultaneously (see Figure 1b, bottom). In this paradigm, different proteins and multiple RNAs are considered simultaneously, allowing for the use of common binding patterns, knowledge transfer between proteins, and ideally, imputation of missing interactions. For instance, this approach can be applied in the context of RNA–protein–DNA triads [9], or more simply for predicting unknown interactions between a given protein and RNA. For instance, methodologies for employing protein and RNA embeddings (ESM2 [19], UniRep [20]) in the context of joint interactome analysis are available.

A multitude of models have been developed to predict RNA-protein interactions [21,22,23,24,25,26,27,28,29,30,31,32,33,34,35]. These models employ diverse approaches within given paradigms. Certain models, for example, catRAPID [32], employ the interaction propensity score and do not rely on machine learning at all. RPI-seq [36] utilizes k-mer counting to generate vector representations of proteins and RNA molecules. The k-mer count vectors of proteins and RNAs are subjected to a series of preprocessing steps and subsequently fed into an SVM or random forest classifier. RPI-seq is trained on a limited dataset derived from PDB RNA–protein complex structures, which limits its capacity for organism-specific predictions. RNACommender [37] is a tool that utilizes a protein sequence and calculates the frequency of specific Pfam domains occurrences. The RNA is vectorized by quantifying subgraphs of the RNA structure predicted by RNAfold [38]. The processing of both vectors is accomplished by linear layers of a neural network with sigmoid activations. The vectors obtained in this manner are subsequently converted into a single value using a trained bilinear form. The sigmoid value of this value is the binding probability.

PrismNet [39] proposes a single-protein approach in which an independent model is constructed for each protein separately. A set of such models enables the enrichment of RNA interactomes based on incomplete experimental data. In summary, RNA sequences are uniformly shortened to equivalent lengths and encoded through the application of one-hot encoding. It is further proposed that an additional channel be utilized to represent the icSHAPE accessibility of each nucleotide. The resulting representation is subsequently fed into a convolutional neural network (CNN) comprising multiple residual blocks and a squeeze-and-excitation block. HDR-Net [40] constitutes an enhanced iteration of PrismNet, characterized by a BERT-inspired representation of nucleotides and a more intricate CNN architecture. Both of these methods employ a benchmark that states PrismNet and HDRNet’s superiority over previously developed methods such as GraphProt [31] and DeepBind [41]. A recent benchmark [42] of RNA–protein interactions prediction models and architectures also points out that there is currently no single method representing a major advantages over the others. The architectures employed in that benchmark also included such models as DeepRiPe [33], Multi-resBind [35] and MultiRBP [34].

However, despite their numerous advantages, both single-protein and multi-protein approaches are highly dependent on the initial eCLIP data, its quality, specificity, and distribution of statistically significant interaction peaks. Despite the existence of established protocols for joint sequential immunoprecipitation [16], the immunoprecipitation technology itself has been the subject of repeated criticism in recent years [13,14,15,43,44]. The eCLIP protocol is subject to systematic biases. First, crosslinking is far from uniform across all RNA regions and is inconsistently shifted with known binding sites of proteins [15]. Second, normalization by transcription level may be incomplete [14]. Third, some of the signals reflect associations of RNA with chromatin-binding proteins or RNA-containing complexes rather than direct binding of the target protein to RNA [13,16]. In this regard, it becomes more attractive to use data on RNA–protein contacts obtained from in vitro experiments, such as SELEX [45] or its alternative RNAcompete [46,47], where the RNA chip design is calibrated so that different RNA k-mers are evenly represented across it.

In this study, we explore the limitations of single- and multi-protein models in predicting RNA–protein interactions. In the case of single-protein models, the features extracted from RNA were limited to sequence information obtained from eCLIP data. Furthermore, when developing multi-protein models, we incorporated protein features obtained from ESM2 and UniRep embeddings for our sample. The findings demonstrate the feasibility of predicting novel interactions between proteins and RNAs of genes with low (practically background-level) expression by single-protein models. We also demonstrate that the task of constructing multi-protein models for de novo prediction of RNA-=protein interactions based on in vivo data may be ill-posed due to the variability of biological aspects of experiments. For instance, the different affinities of the antibodies used to the protein can result in differences in the values of pulldown libraries, and sometimes control libraries as well. Furthermore, we demonstrate that the latent space of RNA–protein contact features which can be determined from in vivo experiments such as eCLIP does not correspond to that obtained from in vitro experiments (RNAcompete).

## 2. Results

The models and their weights obtained during the development of the PLERIO framework are available on the GitHub repository for 220 studied proteins.

### 2.1. Single-Protein Model Performance

**Figure 2:**
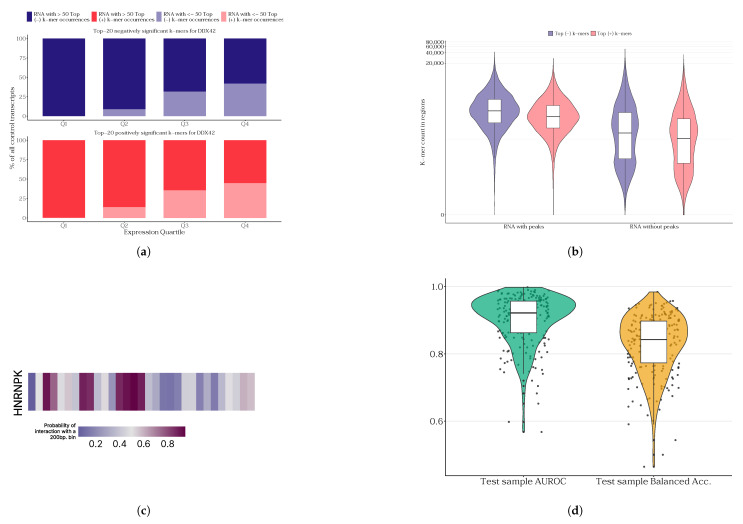
Results of single-protein models developed in the PLERIO framework. (**a**) Enrichment of the top (+) and bottom (−) 20 k-mers identified by the model for DDX42 in transcripts stratified by expression quartiles (Q1–Q4); darker colors indicate the number of transcripts with more than 50 top negative/positive k-mers and lighter shades represent transcripts with 50 or fewer top k-mers. (**b**) Distribution of k-mer enrichment (counts) on RNA with and without protein binding peaks for DDX42. (**c**) Predicted interaction probability track for the HNRNPK protein along the LINC01144 transcript (lowly expressed); bins of 200 bp along the transcript are shown, with color intensity representing the probability of interaction predicted by the model. (**d**) Distribution of quality metrics across test samples for single-protein models.

The primary objective of generating single-protein models is to enhance the RNA interactomes of proteins with existing eCLIP data for lowly expressed RNAs. For each protein, the resulting model was interpreted in the context of k-mers that are significant for predicting the existence or absence of potential interactions with RNA. A comparison was made of the frequency of k-mers significant for the model in highly expressed and lowly expressed transcripts (Figure 2a, with DDX42 protein model shown as a chosen example based on top model performance) and of the presence of such k-mers in statistically significant peaks of protein–RNA interactions (Figure 2b, see also Supplementary Figure S1 for stratified regions and gene length-normalized analysis). It has been demonstrated that RNA molecules with lower expression levels frequently contain a greater number of k-mers that are critical for model prediction. However, the presence of these RNAs in peaks is not guaranteed, as their coverage in the immunoprecipitation experiment is subject to systematic bias due to the lack of normalization for expression levels. Despite the incorporation of background expression level normalization in the ENCODE consortium’s processing pipeline, our findings indicate that this may be insufficient. Though less pronounced, this effect is also conserved when applying normalization by RNA length (see Supplementary Figure S2).

We provide examples of model predictions for some proteins on specific lowly expressed RNAs (see Figure 2c; and see Supplementary Figure S3 for other examples of different proteins and RNAs). For these, we hand-picked lowly expressed RNAs from a sample of protein RNA-seq experiment data and ran the inference for them. Given the potential variability in RNA length and the capacity of the model to only handle sequences up to 200 nucleotides, a screening approach was employed. The model systematically traversed each RNA, with a 200-nucleotide window and a 50-nucleotide step. This approach is more reliable than dividing the RNA sequence into independent segments of 200 nucleotides, as it allows for the spatial proximity of neighboring windows to be taken into account. We note that some of the examples we present fall into peaks of select proteins (e.g., HNRNPK-LINC01144 RNA interaction). However, this is not the case for all the examples we present.

The 220 models we constructed (120 for the K562 cell line and 100 for HepG2) yielded a distribution of prediction quality metrics for RNA–protein interactions in the single-protein model training paradigm (Figure 2d). We also provide the precision–recall curves in Supplementary Figure S4 for the top five best- and worst-performing models. We compare our approach to the SVM-based classifier trained in the same fashion, which serves as a baseline. Our approach outperformed the baseline metrics (Supplementary Figure S5), although our baseline was quite performative itself, consistent with the data reported in a previous benchmark [42]. The quality of our model that bases its predictions solely on RNA sequence data surpasses existing methods for predicting RNA–protein interactions based on deep learning models. This is supported by the observation that the median AUROC distribution of our models exceeds that of competing approaches (Table 1). That said, our proposed approach is also much more straightforward to implement and interpret. The models against which we compare the quality of our predictions utilize more complex approaches to working with RNA sequences, namely, convolutional neural networks (PrismNet) and a composition consisting of a convolutional network with a transformer (HDRNet). Furthermore, it appears that the positive-to-negative class balance in the training sample is a significant factor. Our models were trained on samples where the ratio of positive and negative class samples was 1:1, while PrismNet’s appears to be close to 2:1. It is important to note that although the code and data splits for PrismNet are publicly available, we were unable to reproduce the performance metrics reported by the authors. To avoid introducing bias from our own reproduction attempts of PrismNet and HDRNet, we compare PLERIO’s performance to the metrics reported in the original publications rather than to those obtained in our experiments. The k-mer hashing approach we employed enables us to reduce the embedding dimension of RNA k-mer frequencies up to tenfold without sacrificing quality. Furthermore, using different output embedding sizes did not significantly improve the quality of the predictions for any of the tested proteins (see Supplementary Figure S6). The relative success of hashing raises the question of whether we can effectively compress even larger vectors. Thus, we plan to use hashes to compress k-mer frequency vectors with k>5 in the future.

Another issue to consider is the negative sampling strategy we employed, which may introduce biases into the model’s predictions. To assess whether such biases arise, we stratified model performance on the test set according to three criteria. Specifically, we compared model performance when ground-truth negative samples were stratified by genomic annotation (protein-coding RNAs, lncRNAs, and other RNAs) and by GC content of the corresponding regions. We report the resulting performance metrics in Supplementary Table S2.

We also attempted to cross-reference our findings with independent external datasets. Specifically, we extracted PAR-CLIP, HITS-CLIP, and iCLIP peak sets for the studied proteins from CLIPdb [48] and compared the scores of genomic regions found in eCLIP and external CLIP data predicted by the PLERIO model. The score distributions exhibit an interesting pattern (Supplementary Figure S7): when the overlap between two experiments is large, indicating high consistency between methods for a given protein, the three peak classes (eCLIP-only, external dataset-only, and common peaks) show broadly similar model-based score distributions; in contrast, when the overlap is small, the score distributions for the shared peaks and those unique to the external dataset are comparable, whereas the eCLIP-specific peaks tend to be shifted slightly toward higher (more positive) scores. Overall, the distributions share a similar shape, suggesting that the underlying transcriptomic regions bound by these proteins are largely consistent across immunoprecipitation protocols. However, we note that this analysis is limited by data availability, as it relies on cross-referencing experiments performed in different cell lines (the K562 and HepG2 lines used in our study versus the heterogeneous cell lines represented in CLIPdb).

It should be noted that all currently available in vivo immunoprecipitation-based assays are subject to overlapping sources of technical and experimental bias. To further strengthen external validation, we additionally compare the most informative model-derived k-mers with known sequence motifs obtained from RNAcompete in vitro experiments, which provide a complementary and more independent reference standard. We found a strong positive correlation between our model-derived k-mer scores and the independent RNAcompete z-scores, providing robust external validation (Supplementary Figure S8). For proteins such as HNRNPK, PTBP1, and RBFOX2, a clear positive trend is evident and higher RNAcompete z-scores correspond to higher model scores. This confirms that our model partly recovers binding specificities established in the in vitro assays. Conversely, for other proteins such as FUS, IGF2BP2, and PABPC4 the correlations are weaker or even negative, indicating potential limitations of the model for these specific RNA-binding proteins or inherent differences between in vivo and in vitro binding contexts. Overall, this comprehensive comparison demonstrates that our model’s predictions are biologically meaningful and align well with an independent experimental standard for a significant subset of the analyzed proteins. We must note, however, that CLIP-based methods pose a positive-unlabeled learning problem: the only class we can be certain of consists of the positive examples used to train the model.

Because model training was restricted to sequence features, another sanity check is to assess whether the important k-mers identified by the model correspond to known motifs for the same proteins. To analyze this, we employed a PWM-by-k-mer scoring strategy, in which the position weight matrices (PWMs) of the studied proteins were matched against the top and bottom k-mers from each protein’s model (see Section 4). We then compared the score distributions between top and bottom k-mers to evaluate whether the model captures the sequence-motif principles underlying RNA–protein interactions. We demonstrate (Supplementary Figure S9) that the top-ranked k-mers score significantly higher on known PWMs than bottom-ranked ones, indicating that the model implicitly learns to prioritize sequences matching known RNA-binding motifs. Strong separation is seen for well-characterized RBPs such as PTBP1 and SRSF1; weaker cases such as SSB or ZRANB2 follow the trend, supporting the model’s biological interpretability.

One important caveat to consider is the limitation of the PLERIO framework’s single-protein models. We deliberately excluded RNA features unrelated to primary sequence, although it is well known that some RNA-binding proteins exhibit preferences for RNA secondary structures and spatial folding rather than exact sequence motifs. Several such proteins are included in the set for which we trained models, including SLBP, LIN28B, DGCR8, and ILF3. We report the performance metrics for these models in Supplementary Table S3. Although our models rely exclusively on sequence-based k-mer features, it is noteworthy that the performance of the single-protein models for these structure-associated RBPs is comparable, albeit modestly, to that of other proteins. This observation may be explained by the fact that RNA secondary structures are often partially encoded by local sequence patterns, allowing sequence-based models to capture some aspects of structural preference indirectly.

To demonstrate the applicability of our framework to a real biological task, we assembled a set of proteins that have been experimentally reported to interact with the HIV-1 RNA genome in human cells [49]. Because the model was trained exclusively on K562 or HepG2 genomic data, there is no leakage of HIV transcriptomic sequence into the training process. Therefore, this setup allows us to assess whether the experimentally observed interactions can be computationally recapitulated through machine learning–based inference. We predicted the probabilities of interaction between HIV RNA and each protein and compared these predictions with the annotated regions of the RNA (Supplementary Figure S10). The model predicts that studied RBPs preferentially bind functionally important regions of the HIV-1 genome: 5’ UTR, gag, and env. This aligns with their known roles in RNA processing, translation, and stability. Our findings support the biological plausibility of the model.

### 2.2. Multi-Protein Model Performance

Another objective of the study was to evaluate the feasibility of developing a unified model to predict RNA–protein contacts de novo for proteins for which in vivo immunoprecipitation data have not been produced or published due to various reasons. Creating such a model is of great value, both in itself and for applied tasks; most human RNA-binding proteins lack experimental immunoprecipitation data due to technical infeasibility, making unified de novo prediction models essential to characterize therapeutic targets otherwise inaccessible to conventional methods [50]. One example consists of integrating model predictions with various types of pairwise interaction data [9], such as DNA-protein ChIP-seq data or RNA-chromatin interactome data (Red-C [51], RADICL-seq [52], and GRID-seq [53,54,55]).

**Figure 3:**
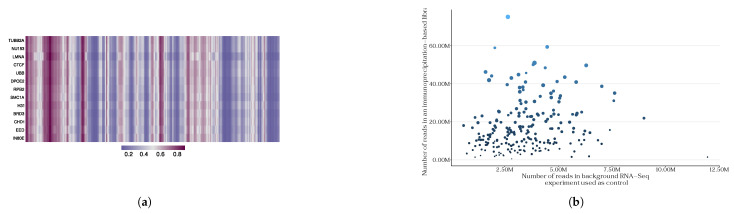
The PLERIO framework multi-protein model. (**a**) An example of predicted interaction tracks for several proteins along the MALAT1 transcript. Each row corresponds to a protein; bins of 200 bp along the transcript are shown, with color intensity representing the probability of interaction predicted by the model. (**b**) The relationship between library sequencing depth and the number of statistically significant RNA–protein interaction peaks in eCLIP experiments. The x-axis represents the number of reads in the background RNA-Seq experiment used as control, while the y-axis shows the number of reads in the immunoprecipitation-based library. Each point corresponds to a protein, with size and color encoding the total eCLIP library size.

To evaluate the specificity of the multiprotein model, we created binding probability tracks for 18 proteins that have various cellular functions and for which eCLIP data are unavailable. Figure 3a shows interaction tracks for selected proteins without publicly available eCLIP data and MALAT1 RNA (see Supplementary Figure S11 for interaction tracks with several different RNAs). Unfortunately, the model’s inability to generalize parameters for de novo imputation of RNA–protein interactions is evident. Perhaps the areas of non-specific activation of the model on MALAT1 for many proteins are regions that could potentially interact electrostatically with proteins, e.g., GC tracts [17]. As expected for a randomly selected set of proteins and random non-coding RNA, no specific binding sites on MALAT1 are observed for all 18 proteins. With corresponding metric values of balanced accuracy (0.79), AUROC (0.87), and MCC (0.52), the model designed in a multi-protein paradigm appears to generate a large number of false positives. This behavior is most likely caused by a scarcity of training data. The model was trained using K562 data with only 90 proteins in the training sample, and 30 were set aside for validation and testing (see Section 4).

It is important to assess whether the model is actually able to generalize in order to determine whether the problem can be addressed using the tools we developed and sequence-only features. We stratified multi-protein model performance into several categories: RNA–protein pairs seen by the model during training, RNA–protein pairs in which either the RNA or the protein was absent during training (i.e., test samples), and RNA–protein pairs that were entirely absent from both training and testing sets. We provide these tracks in Supplementary Figure S12. While the model can generalize to unseen proteins, the quality of this generalization is limited. Consequently, we advise caution when applying PLERIO in this multi-protein setting.

Moreover, a critical component in the construction of this model involves considering the equivalence of the data utilized for its training. The inherent heterogeneity of the biophysical characteristics of eukaryotic proteins presents a challenge when conducting immunoprecipitation of their complexes with RNA, as antibodies for those proteins may exhibit varying affinities. This phenomenon results in disparities in the depth of pulldown sequencing libraries among diverse proteins, consequently yielding varying amounts of training data for different proteins (see Figure 3b). Furthermore, recent studies have raised questions about the biological purity of immunoprecipitation data, and attempts have been made to correct for biological artifacts in such experiments [13,43,44]. As a result, the issue of predicting de novo RNA–protein interactions based on available in vivo eCLIP data alone is either ill-posed or remains infeasible due to the limited amount of currently available data.

### 2.3. eCLIP and RNAcompete Consistency

One emerging domain of research within the broader framework of RNA–protein interactome studies involves the study of in vitro methods and extrapolating their findings to the context of in vivo scenarios. One of the most prevalent in vitro methods is RNAcompete. A thorough analysis of the experimental data has already demonstrated its effectiveness in determining the specificity of binding to motifs in RNA sequences for proteins with or without an RNA-binding domain [17], and has also contributed to the expansion of the range of RNA-protein interactome motifs across the eukaryotic tree of life [47]. To determine the viability of obtaining such results through in vivo experimentation, a similar analysis was conducted on a joint embedding matrix of proteins and their interactomes that was obtained from eCLIP data (see Section 4).

In the joint protein–ligand Embedding (JPLE) approach, the RNA interactome of each specific protein was represented as a vector of 7-mer z-scores retrieved from RNAcompete. We selected a subset of the proteins from the original study for which a public set of eCLIP peaks also existed. To replace the RNAcompete z-score vector, the average signal was normalized across the entire set of 7-mers for those peaks in which each specific 7-mer was present. We then reproduced the singular value decomposition (SVD) analysis of both combined matrices (RNAcompete and eCLIP data) for those proteins.

**Figure 4:**
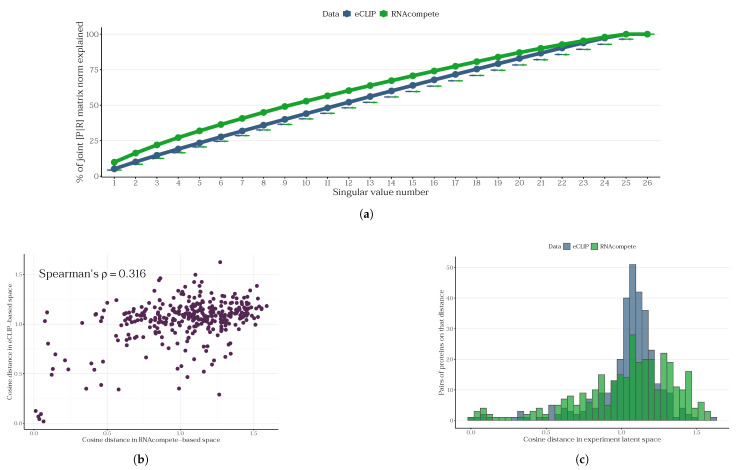
A comparison of the JPLE approach for RNAcompete and eCLIP data. (**a**) The cumulative proportion of the joint protein–RNA matrix norm explained by singular values. The x-axis represents the rank-ordered singular value number (i.e., the number of proteins needed to explain the matrix properies), while the y-axis shows the cumulative percentage of the Frobenius norm of the joint [P|R] matrix explained by the top k singular values. Both datasets exhibit a similar, monotonic increase in explained variance, with RNAcompete consistently explaining slightly more variance than eCLIP at each rank. The boxplots represent a random Gaussian matrix SVD distribution. (**b**) Scatterplot of cosine distances between protein pairs in the eCLIP-based vs. RNAcompete-based latent spaces. Pearson’s r=0.472, Spearman’s ρ=0.316, *p*-value $<10^{-16}$. (**c**) Histograms of the cosine distances within each latent space.

We demonstrate that RNAcompete data provide a more rapid explanation of the variance in RNA–protein interaction data (Figure 4a). The decomposition curve of the RNAcompete-based matrix reaches a plateau faster than than that of the eCLIP data. We also performed a permutation test in which we generated a random matrix with the same norm as the eCLIP- or RNAcompete-based RNA embedding matrix. This provided a background model for an experiment with completely random results. This procedure was repeated 1000 times to generate a large enough sample size; the boxplots are presented in Figure 4a. Because the SVD of a random matrix with a given norm is consistent, the cumulative ratio of the explained norm is proportional to the number of components used.

Furthermore, we investigated the spaces of the latent embeddings into which SVD projects the corresponding matrices. It was observed that the distributions of cosine distances between proteins in these spaces differ between the one constructed based on RNAcompete and the one obtained from eCLIP (Figure 4b,c, Wasserstein’s W1 distance 0.1029).

The sample of proteins in the original JPLE study relied on selecting proteins with canonical RNA-binding domains. Upon incorporating proteins with RNA-binding domains that were not previously considered in JPLE into the specified space, it was observed that the distance distribution remained unchanged (Supplementary Figure S13). Thus, we demonstrate that the underlying biology of in vitro and in vivo experiments differs, and could potentially reflect the faults of eCLIP-based data. We have to note, however, that differences between in vivo and in vitro spaces can reflect both assay artifacts. While RNAcompete provides data on single-protein RNA sequence preferences, the deliberate lack of purification in eCLIP protocol also gives rise to RNA that are only cooperatively bound by the target protein being present in large protein complexes, as well as mismatches on RNA regions that are not the target RBP binding site. Given the mounting criticism in the literature regarding the eCLIP protocol as well as the results reported in this study, it is essential to conduct a thorough analysis to ensure the applicability of eCLIP.

## 3. Discussion

The identification of functional RNA–protein interactions constitutes a pivotal aspect of the investigation of non-coding RNA biology and eukaryotic gene regulatory networks. The expansion and refinement of RNA interactomes of nuclear proteins for which public datasets of in vivo experiments exist is of particular interest. It is evident that protocols designed to identify statistically significant interactions between target proteins are marred by a multitude of experimental (technical) and biological flaws, a fact that has already been recognized and documented within the community. In this study, we critically evaluate the potential for expanding interactomes through the incorporation of low-expression RNAs, which exhibit inadequate coverage in immunoprecipitation pulldown experiments. Additionally, we explore the development of a comprehensive RNA–protein binding model that facilitates de novo prediction of such interactions. This model is particularly essential for the imputation of data concerning proteins that are not represented in public databases due to a lack of experimental data.

In this study, PLERIO, a newly developed framework, was utilized to illustrate the successful incorporation of low-expression RNAs into the RNA-protein interaction interactome within the single-protein model paradigm. The proposed model, which exclusively utilizes RNA sequence properties, has demonstrated superior performance in comparison to previously developed approaches that have employed more intricate neural network architectures. We demonstrate that the adoption of particular preprocessing methodologies such as k-mer hashing [56] can be utilized to simplify the process of training the models.

The interchangeability of promising results obtained from in vitro methods and in vivo immunoprecipitation data was also examined. The results demonstrate that the sequence data obtained from eCLIP defines a protein embedding space that differs from that defined by RNAcompete data. Given the recent advances in high-throughput in vitro profiling (e.g., PRIM-seq [57] and RNAcompete) it is plausible that the next breakthrough in de novo prediction of RNA–protein interactions will stem from leveraging in vitro-derived data. However, it is important to note that these protocols are not without limitations, including the lack of cellular context, oversimplified binding models, an inability to capture cooperative or competitive binding, and a bias toward high-affinity motifs. In this way, we demonstrate that RNA and protein sequence data alone may be insufficient for single base-resolution de novo prediction of RNA–protein interactions from in vivo data. When constructing the model, we deliberately excluded features related to RNA and protein structure.

Moreover, we did not account for the cooperativity of protein binding to different transcripts when constrained by other nuclear proteins. This is a major drawback that must be addressed in future studies; most proteins exhibit different properties when acting as subunits of larger complexes, leading to alterations in their RNA interactome profiles. However, this should be interpreted with caution, as eCLIP-based data inherently represent a mixture of proteins in different states (both as isolated entities and as components of complexes, since no enrichment for single-protein–bound complexes is performed). Future studies should utilize the experimental advantages of methods such as ReCLIP [16] or PRIM-seq [57]. Currently, the PLERIO models presented in this study are agnostic to whether the RNA is bound by a single protein or by a protein within a complex. This may lead to misleading interpretations of the predicted probability of the RNA being bound by the target protein, as this value actually reflects the aggregate probability across all protein states.

Although the current multiprotein mode of PLERIO supports de novo inference on unseen proteins, we strongly discourage its use in this manner. In its present state, this inference mode is prone to both false positives and false negatives, rendering it unreliable for practical applications. We propose that sequence properties may not be the key to predicting interactions de novo, since in contrast to the symmetric task of predicting TF interactions with DNA, the mutual arrangement of RNA and protein pockets in space could play a much more crucial role. Another important parameter to consider is the way to extract protein features from the input protein of interest. Here, we used UniRep [20] and ESM2 [19] embeddings. We selected ESM2 embeddings for the final results based on their superior performance. However, we note that with the advent of new machine learning–based protein representations that incorporate both sequence and 3D-structure–related features, model performance could be significantly improved. We deliberately excluded structure-informed embeddings such as MaSIF [58] as an intentional handicap to assess whether the task could be solved using sequence-only features. Future studies should rigorously evaluate CNN performance on these data and explore the incorporation of protein and RNA features derived from their spatial structures and solvent-accessible surfaces.

Additionally, the function of intrinsically disordered regions (IDRs) within proteins in the context of RNA–protein interactions should be studied. It has been established that a considerable number of nuclear regulatory proteins are endowed with IDRs or are characterized by a complete absence of structure [59,60]. This in turn creates another degree of freedom for RNAs bound to IDR-containing proteins. Current immunoprecipitation protocols do not guarantee that the regions of RNA where protein binding occurs will be true binding sites [15], since interaction peaks in such experiments are often determined by GC-rich sequences due to their electrostatic properties [17]. Future efforts should integrate structural, spatial, and biophysical information alongside high-resolution experimental datasets to achieve a more complete and mechanistic understanding of RNA–protein interactomes. Such integrative approaches will be essential for accurately modeling the complexity of nuclear regulatory networks as well as for extending predictive frameworks to proteins that remain underrepresented.

## 4. Materials and Methods

We selected the datasets encompassing RNA–protein interactions for 220 proteins for this study. In the interest of comprehensively understanding and generalizing the data, it was essential to select the widest possible range of CLIP experiments. To predict RNA–protein data, the PLERIO (Protein-to-Low-Expressed-RNA Interaction Oracle) framework was developed. The source code for this framework is available in the GitHub repository at https://github.com/yotterni/PLERIO.

### 4.1. Data and Preprocessing

We obtained the results of the eCLIP experiments conducted by the ENCODE [11] consortium for 120 proteins in the K562 cell line and 100 proteins in the HepG2 cell line (see Supplementary Table S1 for the identifiers of the ENCODE data used). Because the ENCODE database uses a standardized data processing pipeline, we did not reprocess the data. We took data on statistically significant regions of interaction (IDR-thresholded peaks) between 220 proteins and RNA. Thus, the selected data contain approximately three million unique RNA–protein interaction pairs. For each ENCODE eCLIP experiment, control datasets (RNA-seq corresponding to the pulldown experiment) in BAM format were selected. RNA expression counts were calculated using featureCounts v.2.0.1. RNAcompete data in the form of 7-mer z-scores were obtained from a recent study [47].

We formalized the task of predicting RNA–protein interactions as a binary classification problem. Therefore, the ENCODE data required additional preprocessing. The eCLIP experiment data only contain positive examples of interacting RNA–protein pairs. To train the model, we generated negative examples (non-interacting pairs) as follows: for each protein with known eCLIP results, negative examples were constructed as pairs of the corresponding protein and RNA regions that were not identified as interacting with that protein during the experiment. Thus, either intergenic regions, gene deserts, and introns or regions of RNA that interacted with the protein but for which no peak was detected were selected for the negative sample. To achieve a 1:1 ratio of positive to negative examples in the training sample, a portion of the negative class instances was randomly selected.

The RNA components of RNA–protein pairs were reduced to a uniform size of 200 nucleotides. This fixed input size is required for machine learning models and architectures, and helps to avoid overfitting to RNA length. In order to augment the size of the training sample, a regularization technique was employed in tandem with model augmentation for both positive and negative RNA sequences. A 200-nucleotide window shift to all possible positions from −15 bp downstream to +15 bp upstream was incorporated for positive class, and to all possible positions from −15 bp downstream to +15 bp upstream in increments of 5 bp for negative class.

**Figure 5:**
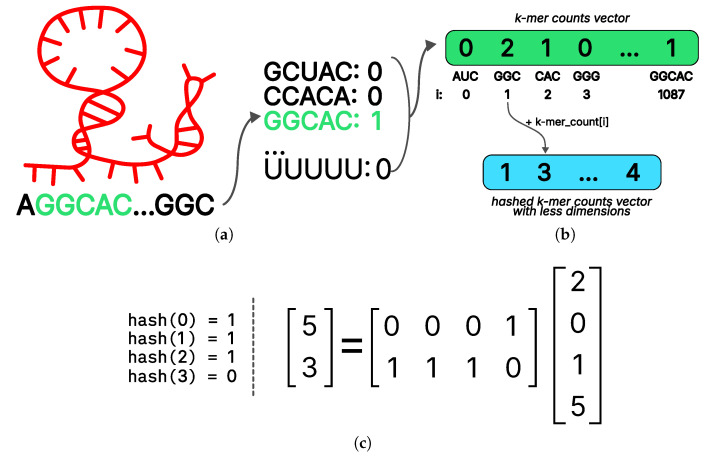
Preprocessing approaches used in the PLERIO framework. (**a**) K-mer counting: A sample RNA sequence (AGGCAC…GGC) is scanned to compute the frequency of all possible 3- and 5-mers; here, GGCAC appears once, while others are absent. (**b**) Vector hashing for dimensionality reduction: the full k-mer count vector is compressed via hashing; each k-mer index is mapped to an output dimension using a hash function, and counts are accumulated into the corresponding bins. (**c**) An example illustrating how a reduction matrix transforms an input vector of dimension 4 into an output of dimension 2. The matrix applies a linear projection to combine input features, compressing the signal while retaining structural relationships.

Given that preprocessed RNA data consist of fixed-length sequences, it is logical to employ standard natural language processing techniques such as recurrent neural networks or transformers for RNA vectorization. However, it should be noted that such models are often trained on sizable datasets. In contrast, we utilized approximately 12,000 examples (including augmentations) for each protein, representing a substantially smaller dataset. As a consequence, we employed a more elementary approach entailing quantification of the frequencies of all k-mers in the RNA (Figure 5a). This approach is reasonably interpretable from a biological perspective [61]. It is well-established that numerous proteins bind to specific RNAs by recognizing short motifs within them. Thus, our proposed vector representation can be regarded as the frequencies of all possible similar patterns of a certain length.

The k values for k-mers length were selected to be 3 and 5. The frequency vectors of 3-mers and 5-mers were concatenated, resulting in a vector representation of RNA with a dimension of $4^{3}+4^{5}=1088$. The selection of sequence size is substantiated by the observation that the models exhibited optimal performance on the validation sample within the specified parameters, while the embedding size remained reasonable.

While the calculation of k-mer frequencies as a means of representing RNA has been demonstrated to be a robust regularization for the model, the dimensionality of the resulting embedding was found to be excessively large. When fed into a linear layer with an output dimension that is half the size of the input, the number of model parameters will exceed the size of the training sample, causing the model to overfit. To mitigate this issue, we employed a hashing method commonly utilized in natural language processing [56] to reduce the dimensionality of the k-dimensional vector.

The first step in the hashing procedure is to determine the desired final dimensionality to be obtained at the conclusion of the procedure. This is followed by the selection of a suitable hash function. In this study, a function from the linear universal family was chosen. Subsequently, for the *i*-th coordinate of the input vector, the value of the hash function hash(i) was calculated and the value of the output vector was incremented in this index; thus, $out\left(hash(i)\right)+=kmer\left(i\right)$ (Figure 5b).

For a fixed hash function, all its values on numbers from 0 to 1087 (as the number of all 3- and 5-dimensions used in the model) can be precalculated in advance. Furthermore, it is feasible to express the hashing procedure in closed form by employing a column-wise stochastic linear operator. An illustration of this procedure with input and output dimensions of 4 and 2, respectively, is presented in Figure 5c. Stochasticity in this context is a property of the operator matrix, indicating that the sum of the values in each column is equal to one. Representing hashing as a simple linear transformation facilitates efficient implementation and enables differentiation by the input vector, a property that can be advantageous for interpreting models built on top of hashing. The ESM2 model [19] was selected as the vector representation of the protein due to the fact that models based on unified representation [20] embedding demonstrated lower quality.

### 4.2. Machine Learning Models

**Figure 6:**
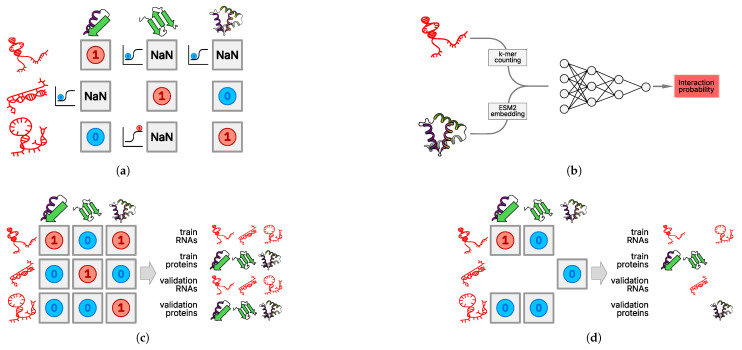
PLERIO framework model data handling. (**a**) Train–test split for single-protein models. The training and test sets are partitioned at the protein level; each RBP has its own interaction matrix, and missing values (NaN) indicate unobserved interactions. (**b**) Multi-protein model architecture. The neural network architecture accepts two inputs: a k-mer counting-embedded RNA sequence, and a protein embedding generated by ESM2. These are concatenated and fed into a fully connected neural network that outputs an interaction probability. (**c**) Naive and (**d**) implemented multi-protein train–test split. We enforced a strict protein-level split in which all interactions of a given protein were assigned exclusively to either the training or validation set. No protein appeared in both sets. This simulates real-world conditions where the model must predict interactions for entirely novel RBPs.

The single-protein paradigm requires training models for each protein. To accomplish this, we selected and preprocessed eCLIP data from the ENCODE database. A model for a single protein receives the features obtained during RNA preprocessing as input and outputs the probability of interaction between that RNA and the corresponding protein (Figure 6a). Such models are necessary for imputing interactions involving lowly expressed RNAs, since it has been shown that information about such RNAs is underrepresented in eCLIP data. eCLIP results are dominated by highly expressed RNAs (see Figure 2a in Section 2), and the statistical processing of experimental data does not include normalization to the level of RNA expression.

For each protein, the set of unique RNAs was randomly divided into training, validation, and test samples. This division was performed independently for the set of unique proteins and RNAs. For a multi-protein model, the protein set was randomly divided. To split the RNA set, the set of all chromosomes was first split, then the RNAs were divided according to their chromosomes of origin. This approach prevents data leakage from the validation and test samples into the training sample, which can occur when the set of unique RNAs is randomly divided into three samples. Because only a fixed-size vector representation of RNA is fed into the single-protein model, ordinary logistic regression is sufficient.

As a baseline, we trained a linear SVM-based classifier (SVC) trained on the same sequence-related RNA features. While the underlying models of SVC and logistic regression are similar, we found the logistic regression-based approach to be more efficient, which is backed by the obtained metrics.

Creating a multi-protein model involves training a single model using RNA and protein features to predict the probability of interaction between an arbitrary RNA and protein. This approach is necessary primarily for predicting the RNA interactome of proteins for which no experimental data are available. The division of the set of RNA–protein pairs appears to be more complex. With a naive random division of unique pairs into three groups, significant data leakage occurs from the training sample into the validation and test samples. To more rigorously evaluate the model, it must receive not just new RNA–protein pairs but pairs of RNAs and proteins that did not occur in the training and test samples separately. Otherwise, data leakage occurs, resulting in an overestimation of the model’s quality (Figure 6c).

The solution to the aforementioned problem is to split the sets of unique RNAs and proteins independently into training, validation, and test samples. For example, to construct the training sample, RNA–protein pairs should be drawn from pairs of “training” RNAs and proteins. This approach results in partial data loss (Figure 6d). Here, we divided the RNAs into three samples using samples of the chromosomes of their origin and randomly divided the set of unique proteins into three groups. Because protein language models accept fixed-size vector representations as input, the vector representation of RNA can be concatenated with it and fed into a fully connected neural network (Figure 6b).

### 4.3. Models Quality Analysis

To assess the performance of our model in terms of external validation sources, we referenced the CLIPdb [48] database, obtaining the RNA–protein peaks generated by immunoprecipitation experiments other than eCLIP. To do this, we filtered experiments that were not “eCLIP” or “RBP_occupancy” in human data, then filtered the results by the proteins we studied. For a single protein immunoprecipitation protocol (e.g., PAR-CLIP), we merged the peaks from different peak-calling software to generate a single dataset of regions to which the protein is bound according to that experiment. We then calculated the scores for peaks in both the reference eCLIP and external PAR-CLIP, HITS-CLIP, and iCLIP datasets if they were available for the studied proteins. We defined the score of a region as the sum of the PLERIO-inferred weights of k-mers present in that region. We scored eCLIP and external protocol peaks for target proteins using the PLERIO-based 3- and 5-mer weights. To understand the effect of the original protocol on the inferred k-mer weights, we stratified the peaks into three groups: peaks common to both eCLIP and another IP protocol, and those exclusive to one of the same two protocols.

We also evaluated the agreement between k-mer–based predictions from our PLERIO model and in vitro binding preferences measured by RNAcompete. For each protein, we projected our 3-mer and 5-mer regression weights onto all possible 7-mers by averaging the scores of overlapping constituent k-mers. We then computed Spearman rank correlations between these predicted 7-mer scores and the corresponding RNAcompete z-scores across the full set of 16,384 7-mers. This genome-wide comparison assessed whether sequence preferences learned from in vivo binding data generalize to orthogonal in vitro measurements.

To assess our models’ capacity to reconstruct the sequence motifs already determined from eCLIP data, we obtained the position weight matrices (PWMs) data from the ATtRACT [62] database. Specifically, for a given PWM, we computed the average probability of observing each k-mer by sliding a window of length k across the PWM, calculating the probability of the k-mer at each position as the product of nucleotide probabilities, and averaging these probabilities across all valid positions. This yields a position-averaged affinity score that reflects the typical binding compatibility of the motif with the k-mer, rather than its total expected abundance. For interpretability, we extracted 5 (out of total 64) 3-mers and 50 (out of total 1024) top and bottom k-mers by model weights.

To assess the performance of the models on a real task, we scored the sequence of the HIV-1 RNA genome (GenBank accession ID: AF033819.3) by models of those proteins reported to interact with it in a previous study [49].

The negative sampling procedure we employed may introduce biases in model predictions for certain classes of cellular transcripts among negative examples. To assess whether this was the case, we evaluated model performance across stratified subsets of the test data. We used three primary stratification criteria: (1) genomic annotation (transcript biotype), (2) GC content of the input sequence, and (3) expression level quartiles derived from control RNA-seq data. Each region’s genetic annotation was obtained from GENCODE [11], and the GC content was binned into four quartiles.

### 4.4. Other Comparative Analyses

To carry out a comparison between in vitro and in vivo data on RNA–protein interactions, a strategy was adapted from a recent study that predicted orthologous RNA–protein interactions in the eukaryotic tree of life [47]. In summary, we reproduced the RNAcompete data processing workflow, constructing a joint embedding matrix of proteins and RNAs followed by SVD with the objective of explaining the RNA profiles matrix norm part of the joint protein–RNA matrix. Furthermore, a comparative analysis was conducted between the latent spaces derived from in vitro and in vivo experimental data using a similar procedure for eCLIP data. We limited ourselves to a list of 26 proteins that had the same RNA-binding domains used in the study, while also retaining a larger list of 56 proteins with a more relaxed RBD definition. Because the original RNAcompete methodology involves scoring the 7-mers, we followed in its tracks and tried scoring the eCLIP peaks similarly. The z-score profile of 7-mers from RNAcompete was substituted with a metric derived from peaks. For each 7-mer, we derived its binding signal by taking the maximum value of fold change times $-\log_{10}$(*p*-value) across all eCLIP peaks where that 7-mer was present. This maximum-value approach was chosen in order to avoid signal averaging effects that could mask genuine binding preferences, as averaging would dilute specific interactions with numerous weaker non-specific binding events commonly observed in protein–DNA interactions. Subsequently, the 7-mer vector was centered and normalized within a single protein, yielding a proxy z-score. Protein embeddings for such a matrix were obtained using methods similar to those in the original study: HMMscan [63] was used to find and then extract target RNA-binding domains from the proteins, and created RNA- and protein-embedding matrices were decomposited. For the extended list of RNA-binding domains used in this study, see Supplementary Table S4.

This and other comparative analyses were conducted using in-house Python 3.10 and shell scripts. Visualization was performed in R 4.5.2. To reproduce the analysis, the scripts can be found in the accompanying GitHub repository at https://github.com/dkhlebn/PLERIO_scripts.

## Figures and Tables

**Table 1:** Median values of the AUROC metric for single-protein classifiers of RNA–protein interactions of PLERIO framework compared against previous approaches. PrismNet and HDRNet metrics were retrieved from the models’ source publications. While the methodologies of both PrismNet and HDRNet are publicly available up to cross-validation fold data, we were unable to reproduce the metrics reported by the authors, yielding lower values.

	PLERIO	PrismNet	HDRNet
median AUROC	0.92	0.85	0.87

## Data Availability

The data that support the findings of this study are available from corresponding author D.A.K. upon reasonable request. PLERIO model weights for proteins in the K562 cell line are available from the Zenodo repository: https://doi.org/10.5281/zenodo.18287748. Scripts for data preprocessing, model training, and inference using the PLERIO framework are available from the GitHub repository: https://github.com/yotterni/PLERIO. Additional scripts for model evaluation, JPLE-like analysis, and k-mer-to-peak analysis are also provided in the GitHub repository: https://github.com/dkhlebn/PLERIO_scripts.

## Supplementary Materials

The following supporting information can be downloaded at: https://www.mdpi.com/article/10.3390/ijms27031192/s1.

## References

[1] Quinn J.J., Chang H.Y. (2016). Unique features of long non-coding RNA biogenesis and function. Nat. Rev. Genet..

[2] Engreitz J.M., Ollikainen N., Guttman M. (2016). Long non-coding RNAs: Spatial amplifiers that control nuclear structure and gene expression. Nat. Rev. Mol. Cell Biol..

[3] Bruel A.L., Vulto-vanSilfhout A.T., Bilan F., Le Guyader G., Gilbert-Dussardier B., Le Guillou X., Rondeau S., Rio M., Lee K.N., Beil A. (2025). Heterozygous CELF4 variants in the N-term region crucial for the RNA-binding activity lead to neurodevelopmental disorder and obesity. Eur. J. Hum. Genet..

[4] Nielsen M., Ulitksy I. (2024). The links are still missing: Revisiting the role of RNA as a guide for chromatin-associated proteins. Mol. Cell.

[5] Ullah I., Thölken C., Zhong Y., John M., Rossbach O., Lenz J., Gößringer M., Nist A., Albert L., Stiewe T. (2022). RNA inhibits dMi-2/CHD4 chromatin binding and nucleosome remodeling. Cell Rep..

[6] Long Y., Hwang T., Gooding A.R., Goodrich K.J., Rinn J.L., Cech T.R. (2020). RNA is essential for PRC2 chromatin occupancy and function in human pluripotent stem cells. Nat. Genet..

[7] Engreitz J.M., Pandya-Jones A., McDonel P., Shishkin A., Sirokman K., Surka C., Kadri S., Xing J., Goren A., Lander E.S. (2013). The Xist lncRNA exploits three-dimensional genome architecture to spread across the X chromosome. Science.

[8] Woo C.J., Kingston R.E. (2007). HOTAIR lifts noncoding RNAs to new levels. Cell.

[9] Khlebnikov D.A., Nikolskaya A.I., Zharikova A.A., Mironov A.A. (2025). Comprehensive analysis of RNA-chromatin, RNA-, and DNA-protein interactions. NAR Genom. Bioinform..

[10] Van Nostrand E.L., Pratt G.A., Shishkin A.A., Gelboin-Burkhart C., Fang M.Y., Sundararaman B., Blue S.M., Nguyen T.B., Surka C., Elkins K. (2016). Robust transcriptome-wide discovery of RNA-binding protein binding sites with enhanced CLIP (eCLIP). Nat. Methods.

[11] ENCODE Project Consortium (2012). An integrated encyclopedia of DNA elements in the human genome. Nature.

[12] Jankowsky E., Harris M.E. (2015). Specificity and nonspecificity in RNA-protein interactions. Nat. Rev. Mol. Cell Biol..

[13] Guo J.K., Blanco M.R., Walkup W.G., Bonesteele G., Urbinati C.R., Banerjee A.K., Chow A., Ettlin O., Strehle M., Peyda P. (2024). Denaturing purifications demonstrate that PRC2 and other widely reported chromatin proteins do not appear to bind directly to RNA in vivo. Mol. Cell.

[14] Kuret K., Amalietti A.G., Jones D.M., Capitanchik C., Ule J. (2022). Positional motif analysis reveals the extent of specificity of protein-RNA interactions observed by CLIP. Genome Biol..

[15] Schwarzl T., Sahadevan S., Lang B., Miladi M., Backofen R., Huber W., Hentze M.W., Tartaglia G.G. (2024). Improved discovery of RNA-binding protein binding sites in eCLIP data using DEWSeq. Nucleic Acids Res..

[16] Ducoli L., Zarnegar B.J., Porter D.F., Meyers R.M., Miao W., Riley N.M., Srinivasan S., Jackrazi L.V., Yang Y.Y., Li Z. (2025). irCLIP-RNP and Re-CLIP reveal patterns of dynamic protein assemblies on RNA. Nature.

[17] Ray D., Laverty K.U., Jolma A., Nie K., Samson R., Pour S.E., Tam C.L., von Krosigk N., Nabeel-Shah S., Albu M. (2023). RNA-binding proteins that lack canonical RNA-binding domains are rarely sequence-specific. Sci. Rep..

[18] Arora V., Sanguinetti G. (2022). Challenges for machine learning in RNA-protein interaction prediction. Stat. Appl. Genet. Mol. Biol..

[19] Lin Z., Akin H., Rao R., Hie B., Zhu Z., Lu W., Smetanin N., Verkuil R., Kabeli O., Shmueli Y. (2023). Evolutionary-scale prediction of atomic-level protein structure with a language model. Science.

[20] Alley E.C., Khimulya G., Biswas S., AlQuraishi M., Church G.M. (2019). Unified rational protein engineering with sequence-based deep representation learning. Nat. Methods.

[21] Wu S., Xu J., Guo J.T. (2025). Accurate prediction of nucleic acid binding proteins using protein language model. Bioinform. Adv..

[22] Liu X., Zhu W., Ding X., Fang Y., Wang S., Zhu L., Shen H.B., Pan X. (2025). Base-resolution binding profile prediction of proteins on RNAs with deep learning. Nucleic Acids Res..

[23] Zhang J., Peng Y., Cui F., Zhang Z., Yan S., Zhang Q. (2025). RMDNet: RNA-aware dung beetle optimization-based multi-branch integration network for RNA-protein binding sites prediction. BMC Bioinform..

[24] Mu Q., Yu G., Zhou G., He Y., Zhang J. (2025). DRBP-EDP: Classification of DNA-binding proteins and RNA-binding proteins using ESM-2 and dual-path neural network. NAR Genom. Bioinform..

[25] Wang Y., Zhu H., Wang Y., Yang Y., Huang Y., Zhang J., Wong K.C., Li X. (2024). EnrichRBP: An automated and interpretable computational platform for predicting and analysing RNA-binding protein events. Bioinformatics.

[26] Wassmer E., Koppány G., Hermes M., Diederichs S., Caudron-Herger M. (2024). Refining the pool of RNA-binding domains advances the classification and prediction of RNA-binding proteins. Nucleic Acids Res..

[27] Roche R., Moussad B., Shuvo M.H., Tarafder S., Bhattacharya D. (2024). EquiPNAS: Improved protein-nucleic acid binding site prediction using protein-language-model-informed equivariant deep graph neural networks. Nucleic Acids Res..

[28] Wang Y., Pan Z., Mou M., Xia W., Zhang H., Zhang H., Liu J., Zheng L., Luo Y., Zheng H. (2023). A task-specific encoding algorithm for RNAs and RNA-associated interactions based on convolutional autoencoder. Nucleic Acids Res..

[29] Wang Y., Chen Z., Pan Z., Huang S., Liu J., Xia W., Zhang H., Zheng M., Li H., Hou T. (2023). RNAincoder: A deep learning-based encoder for RNA and RNA-associated interaction. Nucleic Acids Res..

[30] Arora V., Sanguinetti G. (2022). De novo prediction of RNA-protein interactions with graph neural networks. RNA.

[31] Maticzka D., Lange S.J., Costa F., Backofen R. (2014). GraphProt: Modeling binding preferences of RNA-binding proteins. Genome Biol..

[32] Bellucci M., Agostini F., Masin M., Tartaglia G.G. (2011). Predicting protein associations with long noncoding RNAs. Nat. Methods.

[33] Ghanbari M., Ohler U. (2020). Deep neural networks for interpreting RNA-binding protein target preferences. Genome Res..

[34] Karin J., Michel H., Orenstein Y. MultiRBP. Proceedings of the 12th ACM International Conference on Bioinformatics, Computational Biology, and Health Informatics.

[35] Zhao S., Hamada M. (2021). Multi-resBind: A residual network-based multi-label classifier for in vivo RNA binding prediction and preference visualization. BMC Bioinform..

[36] Muppirala U.K., Honavar V.G., Dobbs D. (2011). Predicting RNA-protein interactions using only sequence information. BMC Bioinform..

[37] Corrado G., Tebaldi T., Costa F., Frasconi P., Passerini A. (2016). RNAcommender: Genome-wide recommendation of RNA–protein interactions. Bioinformatics.

[38] Lorenz R., Bernhart S.H., Höner Zu Siederdissen C., Tafer H., Flamm C., Stadler P.F., Hofacker I.L. (2011). ViennaRNA Package 2.0. Algorithms Mol. Biol..

[39] Xu Y., Zhu J., Huang W., Xu K., Yang R., Zhang Q.C., Sun L. (2023). PrismNet: Predicting protein-RNA interaction using in vivo RNA structural information. Nucleic Acids Res..

[40] Zhu H., Yang Y., Wang Y., Wang F., Huang Y., Chang Y., Wong K.C., Li X. (2023). Dynamic characterization and interpretation for protein-RNA interactions across diverse cellular conditions using HDRNet. Nat. Commun..

[41] Alipanahi B., Delong A., Weirauch M.T., Frey B.J. (2015). Predicting the sequence specificities of DNA- and RNA-binding proteins by deep learning. Nat. Biotechnol..

[42] Horlacher M., Cantini G., Hesse J., Schinke P., Goedert N., Londhe S., Moyon L., Marsico A. (2023). A systematic benchmark of machine learning methods for protein-RNA interaction prediction. Brief. Bioinform..

[43] Lee Y., Das P., Kesner B., Rosenberg M., Blum R., Lee J.T. (2025). Re-analysis of CLAP data affirms PRC2 as an RNA binding protein. bioRxiv.

[44] Guo J.K., Blanco M.R., Guttman M. (2024). Failing to account for RNA quantity inflates background and leads to the misleading appearance that PRC2 and GFP bind to RNA in vivo. bioRxiv.

[45] Tuerk C., Gold L. (1990). Systematic evolution of ligands by exponential enrichment: RNA ligands to bacteriophage T4 DNA polymerase. Science.

[46] Ray D., Kazan H., Chan E.T., Peña Castillo L., Chaudhry S., Talukder S., Blencowe B.J., Morris Q., Hughes T.R. (2009). Rapid and systematic analysis of the RNA recognition specificities of RNA-binding proteins. Nat. Biotechnol..

[47] Sasse A., Ray D., Laverty K.U., Tam C.L., Albu M., Zheng H., Levdansky Y., Lyudovyk O., Dalal T., Nie K. (2025). A resource of RNA-binding protein motifs across eukaryotes reveals evolutionary dynamics and gene-regulatory function. Nat. Biotechnol..

[48] Yang Y.C.T., Di C., Hu B., Zhou M., Liu Y., Song N., Li Y., Umetsu J., Lu Z.J. (2015). CLIPdb: A CLIP-seq database for protein-RNA interactions. BMC Genom..

[49] Knoener R.A., Becker J.T., Scalf M., Sherer N.M., Smith L.M. (2017). Elucidating the in vivo interactome of HIV-1 RNA by hybridization capture and mass spectrometry. Sci. Rep..

[50] Jia Y., Gao B., Tan J., Zheng J., Hong X., Zhu W., Tan H., Xiao Y., Tan L., Cai H. (2026). Deep contrastive learning enables genome-wide virtual screening. Science.

[51] Gavrilov A.A., Zharikova A.A., Galitsyna A.A., Luzhin A.V., Rubanova N.M., Golov A.K., Petrova N.V., Logacheva M.D., Kantidze O.L., Ulianov S.V. (2020). Studying RNA-DNA interactome by Red-C identifies noncoding RNAs associated with various chromatin types and reveals transcription dynamics. Nucleic Acids Res..

[52] Bonetti A., Agostini F., Suzuki A.M., Hashimoto K., Pascarella G., Gimenez J., Roos L., Nash A.J., Ghilotti M., Cameron C.J.F. (2020). RADICL-seq identifies general and cell type–specific principles of genome-wide RNA-chromatin interactions. Nat. Commun..

[53] Li X., Zhou B., Chen L., Gou L.T., Li H., Fu X.D. (2017). GRID-seq reveals the global RNA-chromatin interactome. Nat. Biotechnol..

[54] Li L., Luo H., Lim D.H., Han L., Li Y., Fu X.D., Qi Y. (2021). Global profiling of RNA-chromatin interactions reveals co-regulatory gene expression networks in Arabidopsis. Nat. Plants.

[55] Li J., Xiang Y., Zhang L., Qi X., Zheng Z., Zhou P., Tang Z., Jin Y., Zhao Q., Fu Y. (2022). Enhancer-promoter interaction maps provide insights into skeletal muscle-related traits in pig genome. BMC Biol..

[56] Chen B., Liu Z., Peng B., Xu Z., Li J.L., Dao T., Song Z., Shrivastava A., Re C. MONGOOSE: A Learnable LSH Framework for Efficient Neural Network Training. Proceedings of the International Conference on Learning Representations.

[57] Qi Z., Xue S., Chen J., Zhao W., Johnson K., Wen X., Charles Richard J.L., Lin P., Zhong S. (2025). Genome-wide mapping of RNA-protein associations through sequencing. Nat. Biotechnol..

[58] Gainza P., Sverrisson F., Monti F., Rodolà E., Boscaini D., Bronstein M.M., Correia B.E. (2019). Deciphering interaction fingerprints from protein molecular surfaces using geometric deep learning. Nat. Methods.

[59] Jonas F., Navon Y., Barkai N. (2025). Intrinsically disordered regions as facilitators of the transcription factor target search. Nat. Rev. Genet..

[60] Ginell G.M., Emenecker R.J., Lotthammer J.M., Keeley A.T., Plassmeyer S.P., Razo N., Usher E.T., Pelham J.F., Holehouse A.S. (2025). Sequence-based prediction of intermolecular interactions driven by disordered regions. Science.

[61] Chor B., Horn D., Goldman N., Levy Y., Massingham T. (2009). Genomic DNA k-mer spectra: Models and modalities. Genome Biol..

[62] Giudice G., Sánchez-Cabo F., Torroja C., Lara-Pezzi E. (2016). ATtRACT—a database of RNA-binding proteins and associated motifs. Database.

[63] Madeira F., Madhusoodanan N., Lee J., Eusebi A., Niewielska A., Tivey A.R.N., Lopez R., Butcher S. (2024). The EMBL-EBI Job Dispatcher sequence analysis tools framework in 2024. Nucleic Acids Res..

