# Supplementary material for: Sequence-Based Models for RNA–Protein Interactions Imputation Might Be Insufficient for Novel Signal Prediction in eCLIP Data
Source: Int J Mol Sci. 2026 Jan 24;27(3):1192. doi: 10.3390/ijms27031192 (PMC12898063; doi:10.3390/ijms27031192)
Supplement: Supplementary file 1 [file ijms-27-01192-s001.zip › ijms-4091964-supplementary.pdf]

Supplementary Materials

Table S1: PLERIO single-protein models performance for proteins preferring structural motifs of RNA molecules. We note that SLBP metrics are subject to doubt because only 160 peaks of that protein were available in ENCODE. 2
Table S2: Median performance metrics for PLERIO across stratifications. 3
Table S3: ENCODE dataset identifiers for data used in the study. 4
Table S4: Extended RNA-binding domains list used for this study. 11
Figure S1: Stratified analysis of DDX42 model k-mer importance correlation with the k-mer being the statistically significant peak of the DDX42-RNA interaction: **A** figure from original manuscript; **B** same, but the number of important k-mers was normalized to total region length on a transcript; **C** number of important k-mers on transcripts with or without peaks on them, if and RNA has a peak on it, it is split into peaks and regions outside the peaks; **D** same, but normalized to the according region's legth transcript-wise. 13
Figure S2: Enrichment of significant k-mers identified by the model based for DDX42 protein in highly and lowly expressed transcripts, normalized by gene length. 13
Figure S3: Examples of single-protein models ncRNA-protein contact inference in PLERIO framework: **A** CPEB-DT and CPEB4 protein, possible family-wise regulation; **B** THOC7-AS1 and NCBP2 protein, possibly connected via RNA export; **C** GLIS2-AS1 and RBFOX2 protein, possibly connected through involvement in splicing control; **D** NDUFB2-AS1 RNA and SAFB and SAFB2 proteins, possible antisense RNA interaction; **E** PRKAR2A-AS1 and NONO protein, possibly interacting in paraspeckles; **F** RN7SL23P RNA and SSB and TROVE2 proteins. . . . 14
Figure S4: Precision-Recall curves for top-5 best (GTF2F1, HNRNPK, XRN2, FTO, AKAP8L) and worst (NSUN2, WDR3, GNL3, RPS11, UTP3) performing models. 15
Figure S5: Single-protein baseline linear SVC performance compared to PLERIO model performance. **A** Distribution of balanced accuracy and AUROC metrics for the SVC classifier for each of the single-protein models, **B** scatterplot of PLERIO and SVC metrics comparison for each of the models. 15
Figure S6: The effects of varying k-mer hasing output vector dimension on model performance. 15
Figure S7: PLERIO-based scoring of eCLIP peaks compared to other CLIPdb datasets: PAR-CLIP, HITS-CLIP, iCLIP. Three groups of intervals are scored: "common" - peaks found in both datasets, "eCLIP_only" - peaks found exclusively in eCLIP peaks set and "external only" - peaks found only in external set of peaks for a given protein. See captions of images for protein gene symbol and the dataset that eCLIP data is being compared to. 18
Figure S8: Correlation analysis of RNAcompete-based 7-mer z-scores and PLERIO-inferred model scores obtained from 3- and 5-mers. **A** correlation for each of the proteins studied that have both RNAcompete and eCLIP assay data on K562 cell line, **B** scatterplots for RNAcompete 7-mers z-score and PLERIO-inferred eCLIP-based 7-mer score. 18
Figure S9: PLERIO-inferred k-mers scored by each of the proteins PWMs. Top and bottom 3- and 5-mers from each protein models were scored against a corresponding set of PWMs obtained from ATtRACT database. 19
Figure S10: PLERIO-inferred probabilities of select proteins to bind HIV-1 RNA. The green track shows probability for sliding window of size 200 bp and a step of 50 bp. Gray track represents the quantized probability of interaction and is equal to 1 if the probability is larger that 0.5. 20
Figure S11: Examples of multi-protein model ncRNA-protein contact inference in PLERIO framework: **A** NEAT1; **B** HOTAIR; **C** TERC; with multiple proteins covering a wide range of functions where RNA-binding might not be needed. Yet, the interaction profile is practically the same. 21
Figure S12: Examples of multi-protein model prediction for MALAT1 RNA stratified by a sample of protein: proteins are either from training, testing or independent sets. 22
Figure S13: Distribution of cosine distances in eCLIP-based latent space obtained by JPLE algorithm on available proteins with a canonical RBD or a domain that can be less strictly considered RNA-binding. Wasserstein's W1 distance between two distributions is 0.01697. 23

Supplementary Table S1. PLERIO single-protein models performance for proteins preferring structural motifs of RNA molecules. We note that SLBP metrics are subject to doubt because only 160 peaks of that protein were available in ENCODE.

Protein	AUROC	Average precision	Accuracy	Balanced accuracy	F1	Precision	Recall
DGCR8	0.903	0.875	0.817	0.825	0.805	0.737	0.886
ILF3	0.805	0.827	0.730	0.727	0.751	0.749	0.754
LIN28B	0.848	0.845	0.764	0.764	0.763	0.772	0.755
SLBP	0.819	0.639	0.724	0.629	0.333	0.250	0.500

Supplementary Table S2. Median performance metrics for PLERIO across stratifications.

**Stratification**	**Group**	**PLERIO**	
		**AUROC**	**Balanced Acc.**
**Annotation**	protein coding	0.833	0.761
	lncRNAs	0.837	0.789
	other	0.955	0.868
**GC content**	Q1	0.843	0.757
	Q2	0.875	0.650
	Q3	0.500	0.500
	Q4	0.974	0.500
**Expression**	Q1	0.756	0.602
	Q2	0.749	0.667
	Q3	0.805	0.663
	Q4	0.694	0.559

Supplementary Table S3. ENCODE dataset identifiers for data used in the study.

Protein	Cell line	eCLIP ENCODE accession
AARS	K562	ENCFF128BDG
AATF	K562	ENCFF955LYD
ABCF1	K562	ENCFF941GJK
AGGF1	HepG2	ENCFF992LCW
AGGF1	K562	ENCFF810NKV
AKAP1	HepG2	ENCFF749IUB
AKAP1	K562	ENCFF033EFV
AKAP8L	K562	ENCFF205UTG
APOBEC3C	K562	ENCFF472CCL
AQR	HepG2	ENCFF323PUC
AQR	K562	ENCFF949QGM
BCCIP	HepG2	ENCFF134LIU
BCLAF1	HepG2	ENCFF810EUA
BUD13	HepG2	ENCFF011NAP
BUD13	K562	ENCFF262ILV
CDC40	HepG2	ENCFF027MEO
CPEB4	K562	ENCFF665BGJ
CPSF6	K562	ENCFF635NZZ
CSTF2	HepG2	ENCFF349SVY
CSTF2T	HepG2	ENCFF141DOI
CSTF2T	K562	ENCFF878VVA
DDX21	K562	ENCFF378OYA
DDX24	K562	ENCFF537KWB
DDX3X	HepG2	ENCFF777NMK
DDX3X	K562	ENCFF087MAJ
DDX42	K562	ENCFF399GGH
DDX51	K562	ENCFF799ERK
DDX52	HepG2	ENCFF005GER
DDX52	K562	ENCFF198ULC

See next page

Protein	Cell line	eCLIP ENCODE accession
DDX55	HepG2	ENCFF797QRS
DDX55	K562	ENCFF462WAI
DDX59	HepG2	ENCFF899AWZ
DDX6	HepG2	ENCFF614NUP
DDX6	K562	ENCFF904KTV
DGCR8	HepG2	ENCFF193URO
DGCR8	K562	ENCFF023YVD
DHX30	HepG2	ENCFF663QIZ
DHX30	K562	ENCFF128AKC
DKC1	HepG2	ENCFF633LQC
DROSHA	HepG2	ENCFF854BHP
DROSHA	K562	ENCFF797BHU
EFTUD2	HepG2	ENCFF796RMF
EFTUD2	K562	ENCFF862ZPV
EIF3D	HepG2	ENCFF056UAW
EIF3G	K562	ENCFF634UAC
EIF3H	HepG2	ENCFF813JIK
EIF4G2	K562	ENCFF207BXK
EWSR1	K562	ENCFF607ZRF
EXOSC5	HepG2	ENCFF862EKD
EXOSC5	K562	ENCFF858CSS
FAM120A	HepG2	ENCFF208ZMI
FAM120A	K562	ENCFF158AOV
FASTKD2	HepG2	ENCFF498IGK
FASTKD2	K562	ENCFF423SOR
FKBP4	HepG2	ENCFF957AOZ
FMR1	K562	ENCFF480NUZ
FTO	HepG2	ENCFF351ZDU
FTO	K562	ENCFF924OVC

See next page

Protein	Cell line	eCLIP ENCODE accession
FUBP3	HepG2	ENCFF466UMQ
FUS	HepG2	ENCFF972DFZ
FUS	K562	ENCFF861KMV
FXR1	K562	ENCFF366UJP
FXR2	HepG2	ENCFF112MVG
FXR2	K562	ENCFF315HYU
G3BP1	HepG2	ENCFF899HHP
GEMIN5	K562	ENCFF050VVI
GNL3	K562	ENCFF048RLZ
GPKOW	K562	ENCFF674BUM
GRSF1	HepG2	ENCFF929AWR
GRWD1	HepG2	ENCFF159MMZ
GRWD1	K562	ENCFF327DLL
GTF2F1	HepG2	ENCFF002HYO
GTF2F1	K562	ENCFF278ZCU
HLTF	HepG2	ENCFF048FDF
HLTF	K562	ENCFF406MSI
HNRNPA1	HepG2	ENCFF797GSK
HNRNPA1	K562	ENCFF392AEV
HNRNPC	HepG2	ENCFF440ROZ
HNRNPC	K562	ENCFF167CDB
HNRNPK	HepG2	ENCFF855CPQ
HNRNPK	K562	ENCFF918XJQ
HNRNPL	HepG2	ENCFF266TKW
HNRNPL	K562	ENCFF917CBK
HNRNPM	HepG2	ENCFF752JNY
HNRNPM	K562	ENCFF445ENC
HNRNPU	HepG2	ENCFF039XQD
HNRNPU	K562	ENCFF241DVU

See next page

Protein	Cell line	eCLIP ENCODE accession
HNRNPUL1	HepG2	ENCFF610JVZ
HNRNPUL1	K562	ENCFF889AWX
IGF2BP1	HepG2	ENCFF442USD
IGF2BP1	K562	ENCFF650LMV
IGF2BP2	K562	ENCFF524ZZB
IGF2BP3	HepG2	ENCFF886SDQ
ILF3	HepG2	ENCFF071QDP
ILF3	K562	ENCFF385MJU
KHDRBS1	K562	ENCFF027UTH
KHSRP	HepG2	ENCFF771QAU
KHSRP	K562	ENCFF031FMO
LARP4	HepG2	ENCFF534JCV
LARP4	K562	ENCFF947CIN
LARP7	HepG2	ENCFF307MDW
LARP7	K562	ENCFF809CKN
LIN28B	HepG2	ENCFF341XMP
LIN28B	K562	ENCFF061XNA
LSM11	HepG2	ENCFF985ECA
LSM11	K562	ENCFF858EHN
MATR3	HepG2	ENCFF587KKM
MATR3	K562	ENCFF246EPM
METAP2	K562	ENCFF644LPB
MTPAP	K562	ENCFF883KCN
NCBP2	HepG2	ENCFF692RZM
NCBP2	K562	ENCFF886MLH
NIP7	HepG2	ENCFF033MGP
NIPBL	K562	ENCFF554NIV
NKRF	HepG2	ENCFF045HSV
NOL12	HepG2	ENCFF914WFQ

See next page

**Protein**	**Cell line**	**eCLIP ENCODE accession**
NOLC1	HepG2	ENCFF137YFD
NOLC1	K562	ENCFF327YTD
NONO	K562	ENCFF730QRI
NPM1	K562	ENCFF154AYH
NSUN2	K562	ENCFF233PRB
PABPC4	K562	ENCFF452MJL
PABPN1	HepG2	ENCFF709FEU
PCBP1	HepG2	ENCFF098REE
PCBP1	K562	ENCFF900IOH
PCBP2	HepG2	ENCFF642GNE
PHF6	K562	ENCFF588ITW
POLR2G	HepG2	ENCFF591BUK
PPIG	HepG2	ENCFF986XOC
PPIL4	K562	ENCFF559HGK
PRPF4	HepG2	ENCFF227EJF
PRPF8	HepG2	ENCFF048YPA
PRPF8	K562	ENCFF858UKE
PTBP1	HepG2	ENCFF726SQU
PTBP1	K562	ENCFF907HNN
PUM1	K562	ENCFF094MQV
PUM2	K562	ENCFF880MWQ
PUS1	K562	ENCFF247NXL
QKI	HepG2	ENCFF704OCI
QKI	K562	ENCFF786UOW
RBFOX2	HepG2	ENCFF871NYM
RBFOX2	K562	ENCFF206RIM
RBM15	HepG2	ENCFF054VLU
RBM15	K562	ENCFF597MMG
RBM22	HepG2	ENCFF293IZG

See next page

Protein	Cell line	eCLIP ENCODE accession
RBM22	K562	ENCFF972ZMJ
RBM5	HepG2	ENCFF927KRA
RPS11	K562	ENCFF313WDF
RPS3	HepG2	ENCFF301IJW
RPS3	K562	ENCFF530HTL
SAFB	HepG2	ENCFF232WUE
SAFB	K562	ENCFF953WTP
SAFB2	K562	ENCFF594EDV
SBDS	K562	ENCFF051EEW
SDAD1	HepG2	ENCFF825UVD
SDAD1	K562	ENCFF114EUH
SERBP1	K562	ENCFF295HRZ
SF3A3	HepG2	ENCFF950VZO
SF3B1	K562	ENCFF887ARJ
SF3B4	HepG2	ENCFF073IJF
SF3B4	K562	ENCFF649GEE
SFPQ	HepG2	ENCFF139NXB
SLBP	K562	ENCFF623WGE
SLTM	HepG2	ENCFF121RVH
SLTM	K562	ENCFF696QWE
SMNDC1	HepG2	ENCFF736RYV
SMNDC1	K562	ENCFF943XOU
SND1	HepG2	ENCFF609LWQ
SND1	K562	ENCFF211TRO
SRSF1	HepG2	ENCFF934ANS
SRSF1	K562	ENCFF886XUO
SRSF7	HepG2	ENCFF317HGW
SRSF7	K562	ENCFF780OCY
SRSF9	HepG2	ENCFF765PIF

See next page

Protein	Cell line	eCLIP ENCODE accession
SSB	HepG2	ENCFF848JWA
SSB	K562	ENCFF328KJW
STAU2	HepG2	ENCFF678FCX
SUB1	HepG2	ENCFF335JTC
SUGP2	HepG2	ENCFF191XRG
SUPV3L1	HepG2	ENCFF999ZAW
SUPV3L1	K562	ENCFF239GPP
TAF15	HepG2	ENCFF566EAE
TAF15	K562	ENCFF822NWY
TARDBP	K562	ENCFF037TVC
TBRG4	HepG2	ENCFF869RUC
TBRG4	K562	ENCFF618WYU
TIA1	HepG2	ENCFF759KCD
TIA1	K562	ENCFF918KMT
TIAL1	HepG2	ENCFF612HOP
TRA2A	HepG2	ENCFF766OCH
TRA2A	K562	ENCFF726PFJ
TROVE2	HepG2	ENCFF611SOO
TROVE2	K562	ENCFF794RJQ
U2AF1	HepG2	ENCFF056LEW
U2AF1	K562	ENCFF640IHY
U2AF2	HepG2	ENCFF721PWF
U2AF2	K562	ENCFF290DFO
UCHL5	HepG2	ENCFF573DXD
UCHL5	K562	ENCFF365LFU
UPF1	HepG2	ENCFF687EQE
UPF1	K562	ENCFF597NVR
UTP18	HepG2	ENCFF644EIR
UTP18	K562	ENCFF783INO

See next page

Supplementary Table S3 – continued from previous page

Protein	Cell line	eCLIP ENCODE accession
UTP3	K562	ENCFF028GAJ
WDR3	K562	ENCFF560RWD
WDR43	HepG2	ENCFF945NFS
WDR43	K562	ENCFF244JER
WRN	K562	ENCFF404HHM
XPO5	HepG2	ENCFF327VCE
XRCC6	HepG2	ENCFF993VRZ
XRCC6	K562	ENCFF958GMP
XRN2	HepG2	ENCFF321HPH
XRN2	K562	ENCFF695OLO
YBX3	HepG2	ENCFF185OEI
YBX3	K562	ENCFF746XLE
YWHAG	K562	ENCFF932UXC
ZC3H11A	HepG2	ENCFF592WIH
ZC3H11A	K562	ENCFF258HOX
ZC3H8	K562	ENCFF382URH
ZNF622	K562	ENCFF285IGP
ZNF800	HepG2	ENCFF343TCO
ZNF800	K562	ENCFF105HMJ
ZRANB2	K562	ENCFF058OOW

Supplementary Table S4. Extended RNA-binding domains list used for this study.

Domain mnemonic
NHL
SAM_1
KH_1
KH_2
CSD
DSRM

See next page

Supplementary Table S4 – continued from previous page

**Domain mnemonic**
La
PUF
S1
YTH
zf-CCCH
zf-CCHC
zf-CCHH
zf-RanBP
DEAD
Helicase_C
Piwi
Tudor
OB
G-patch
Pumilio_1
RNase_H
RNase_P
TruB_N
LARP_1
U2AF
SF1
PTB
hnRNP

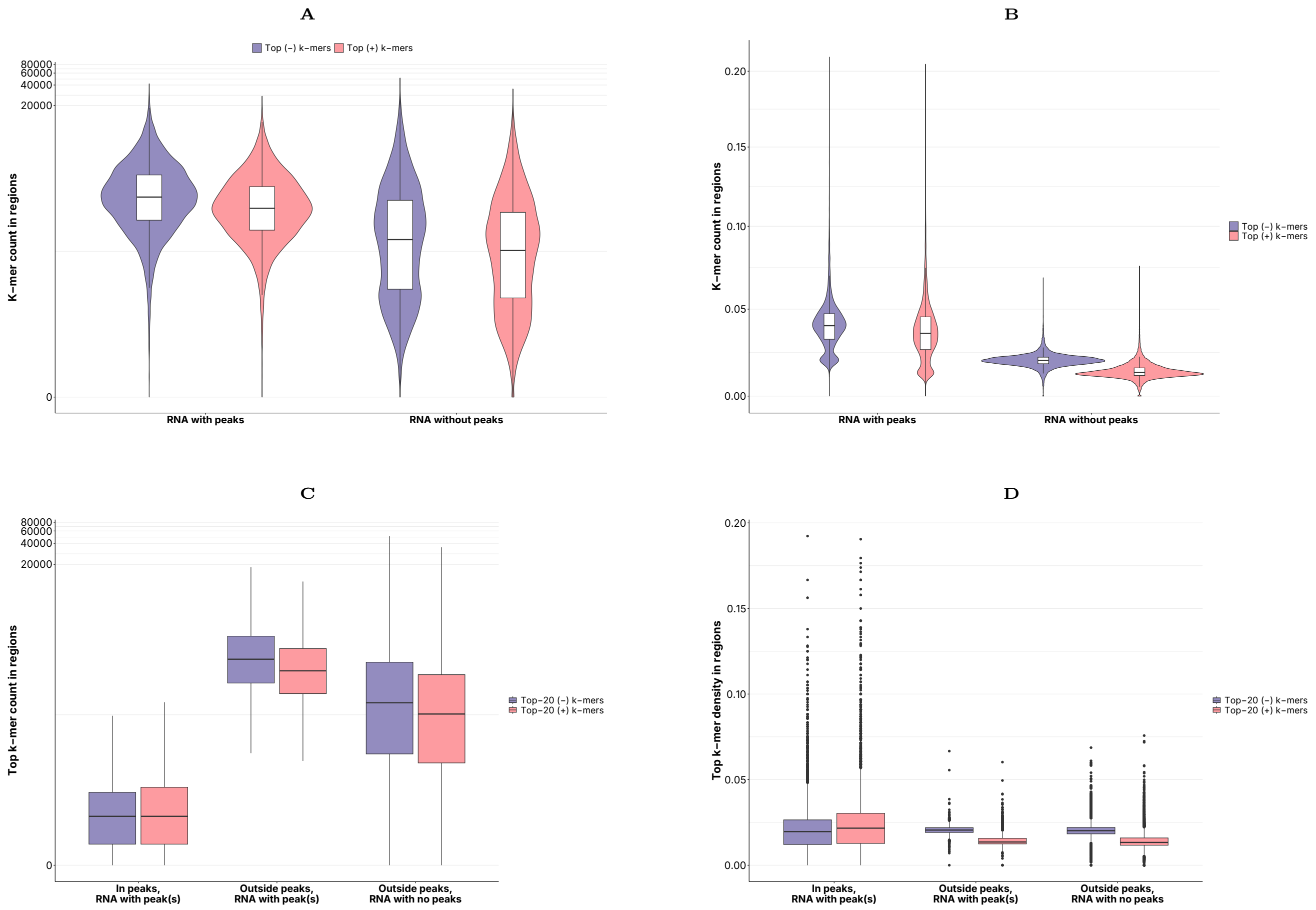

Supplementary Figure S1. Stratified analysis of DDX42 model k-mer importance correlation with the k-mer being the statistically significant peak of the DDX42-RNA interaction: **A** figure from original manuscript; **B** same, but the number of important k-mers was normalized to total region length on a transcript; **C** number of important k-mers on transcripts with or without peaks on them, if and RNA has a peak on it, it is split into peaks and regions outside the peaks; **D** same, but normalized to the according region's legth transcript-wise.

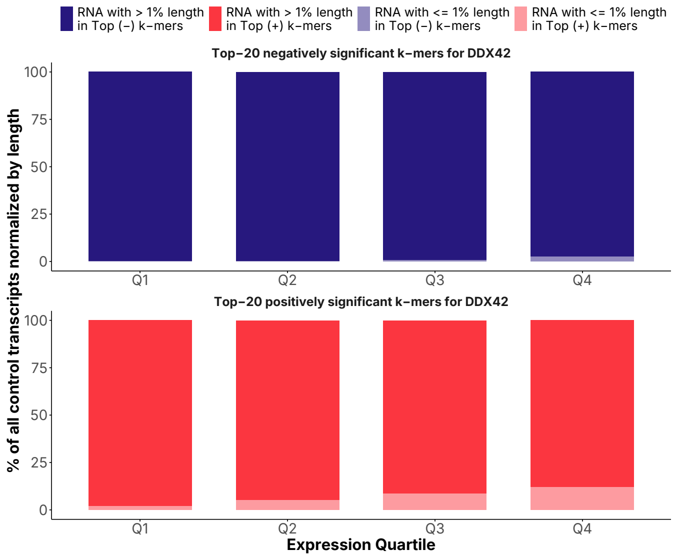

Supplementary Figure S2. Enrichment of significant k-mers identified by the model based for DDX42 protein in highly and lowly expressed transcripts, normalized by gene length.

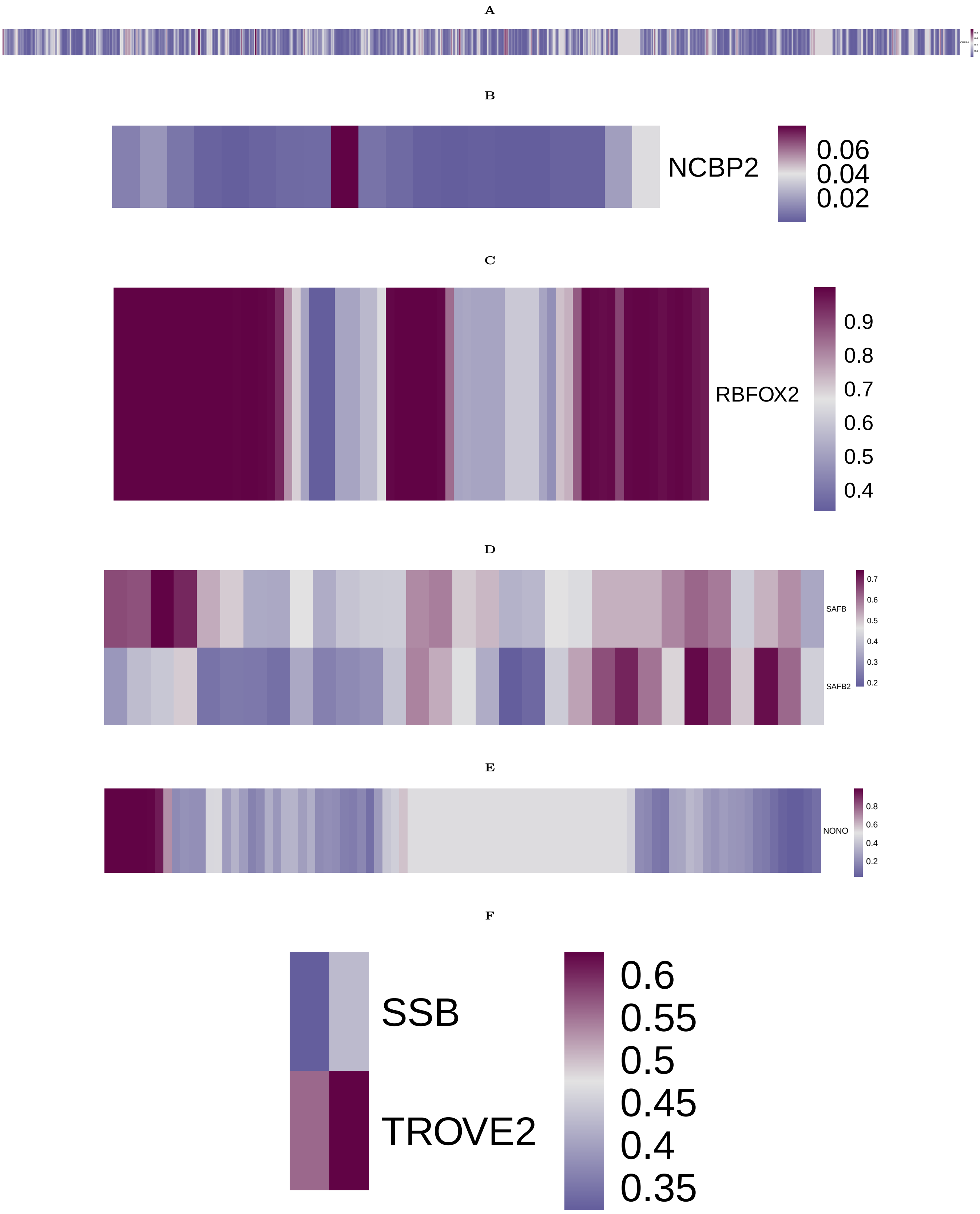

Supplementary Figure S3. Examples of single-protein models ncRNA-protein contact inference in PLERIO framework: **A** CPEB-DT and CPEB4 protein, possible family-wise regulation; **B** THOC7-AS1 and NCBP2 protein, possibly connected via RNA export; **C** GLIS2-AS1 and RBFOX2 protein, possibly connected through involvement in splicing control; **D** NDUFB2-AS1 RNA and SAFB and SAFB2 proteins, possible antisense RNA interaction; **E** PRKAR2A-AS1 and NONO protein, possibly interacting in paraspeckles; **F** RN7SL23P RNA and SSB and TROVE2 proteins.

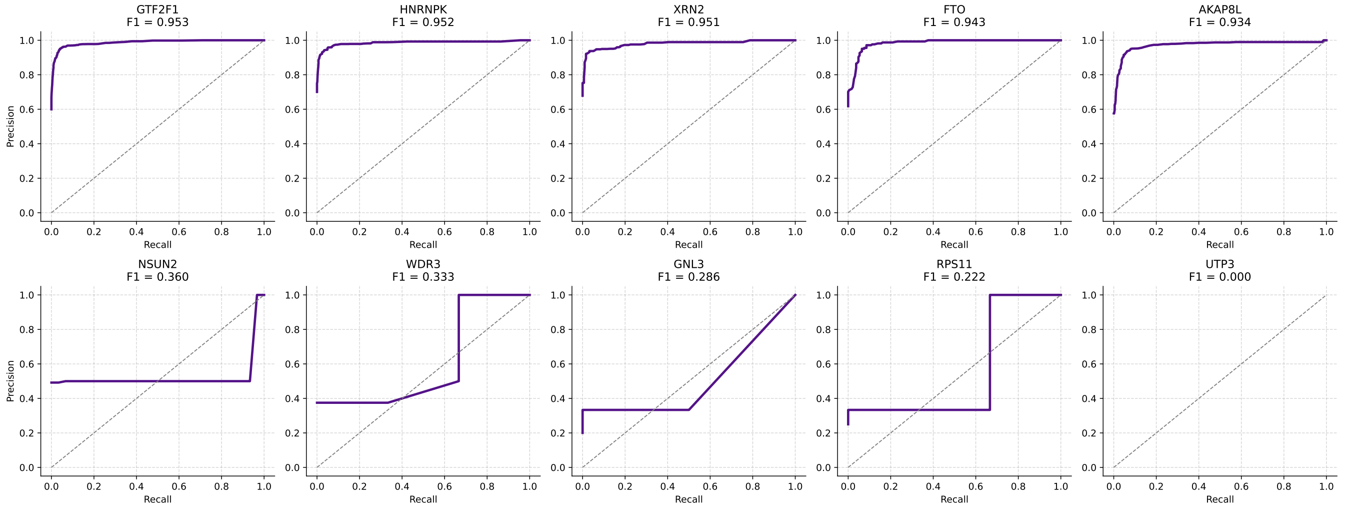

Supplementary Figure S4. Precision-Recall curves for top-5 best (GTF2F1, HNRNPK, XRN2, FTO, AKAP8L) and worst (NSUN2, WDR3, GNL3, RPS11, UTP3) performing models.

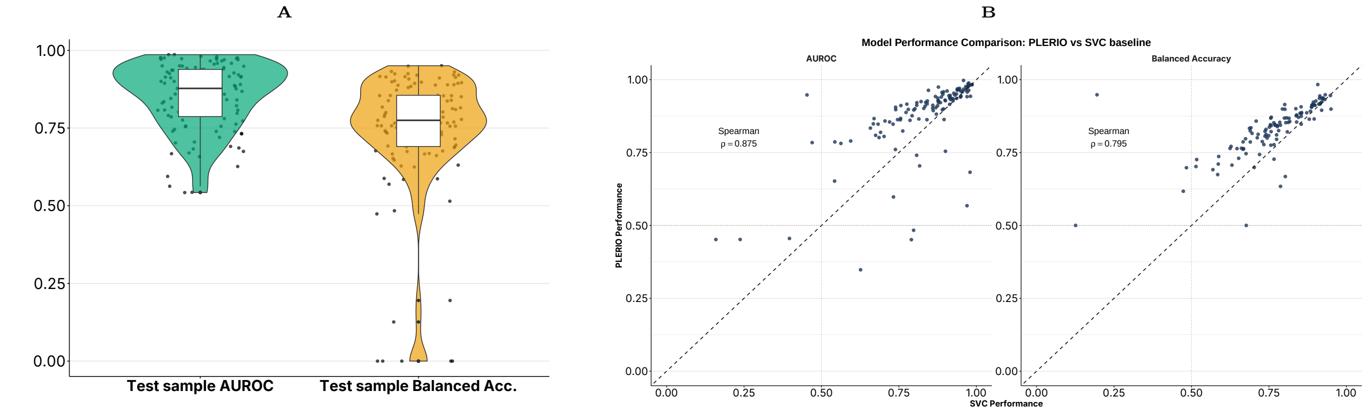

Supplementary Figure S5. Single-protein baseline linear SVC performance compared to PLERIO model performance. **A** Distribution of balanced accuracy and AUROC metrics for the SVC classifier for each of the single-protein models, **B** scatterplot of PLERIO and SVC metrics comparison for each of the models.

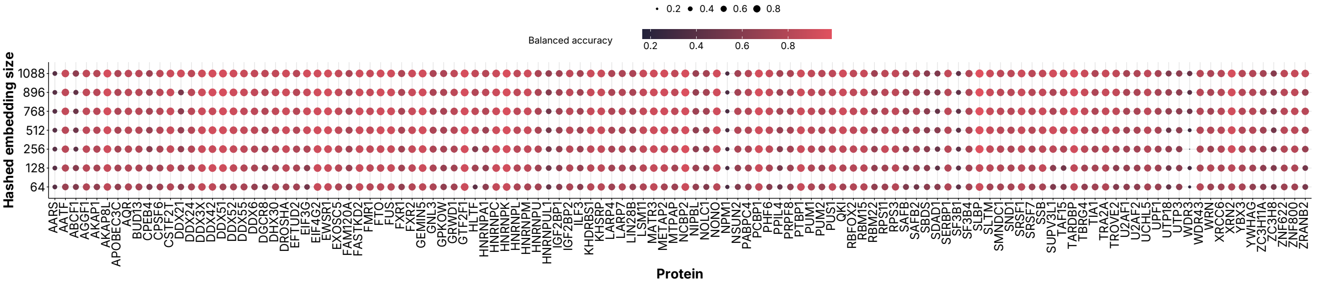

Supplementary Figure S6. The effects of varying k-mer hasing output vector dimension on model performance.

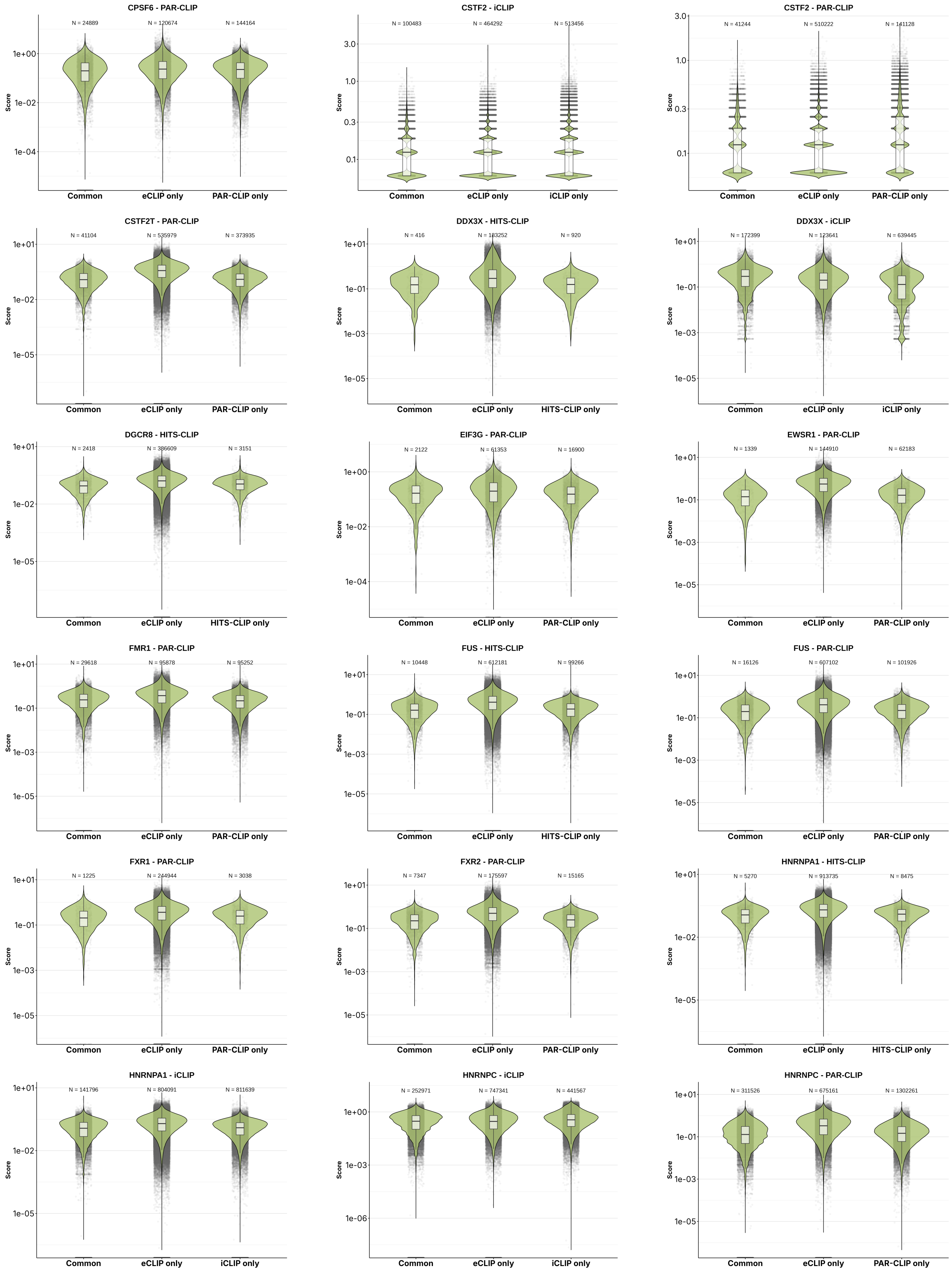

See next page for caption

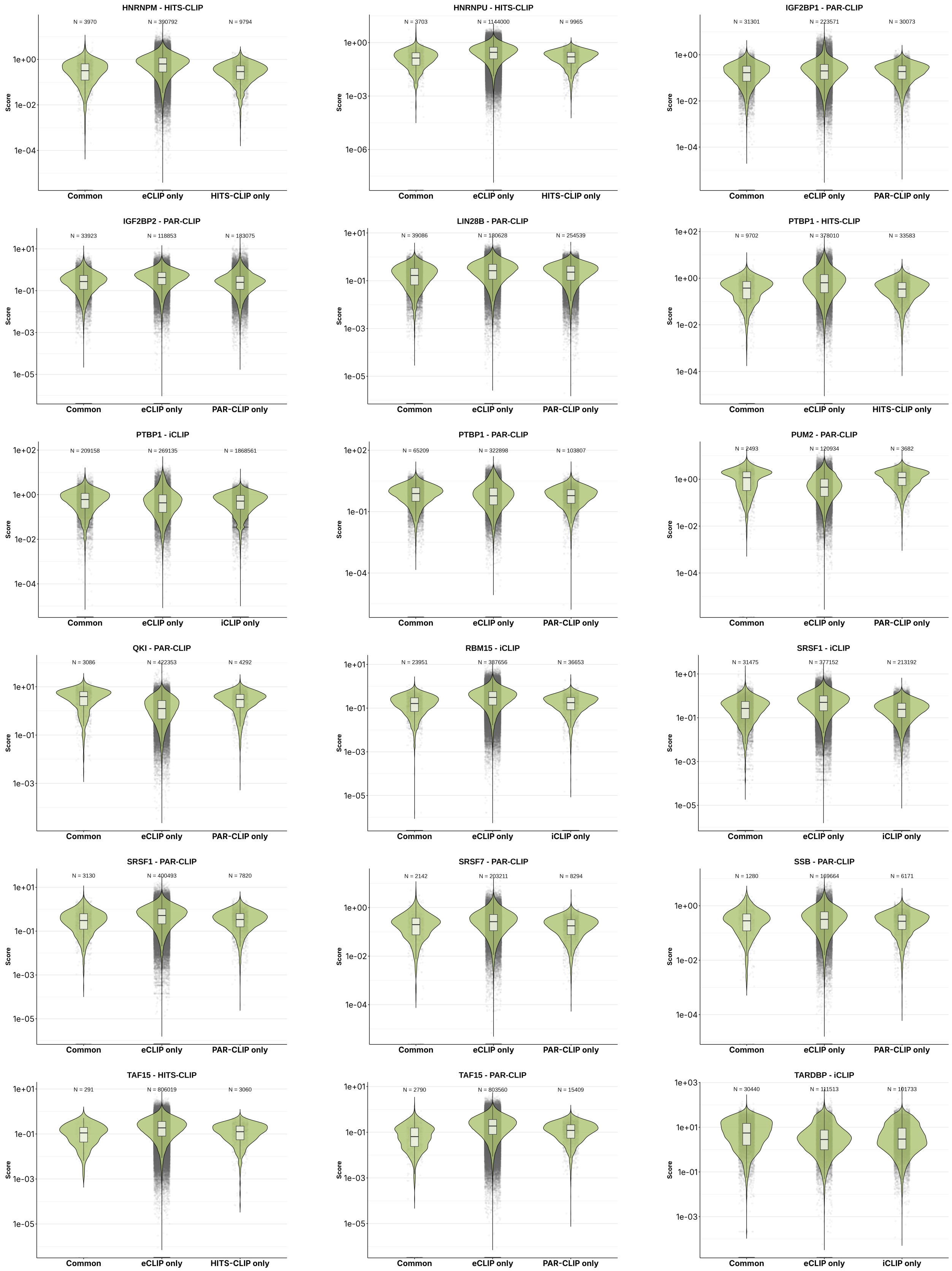

See next page for caption

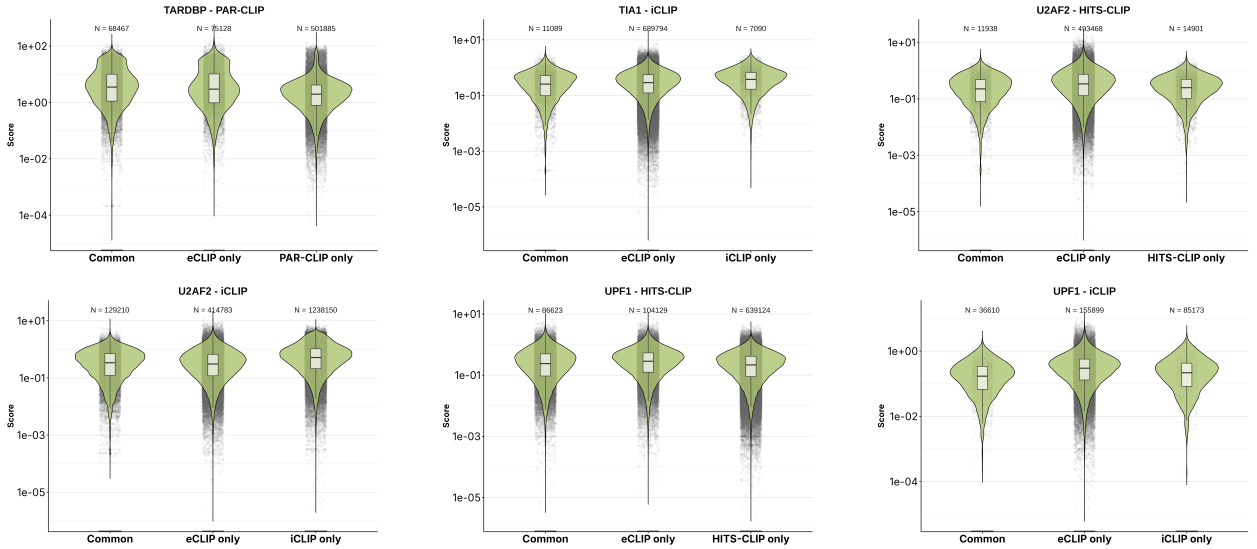

Supplementary Figure S7. PLERIO-based scoring of eCLIP peaks compared to other CLIPdb datasets: PAR-CLIP, HITS-CLIP, iCLIP. Three groups of intervals are scored: "common" - peaks found in both datasets, "eCLIP_only" - peaks found exclusively in eCLIP peaks set and "external only" - peaks found only in external set of peaks for a given protein. See captions of images for protein gene symbol and the dataset that eCLIP data is being compared to.

A

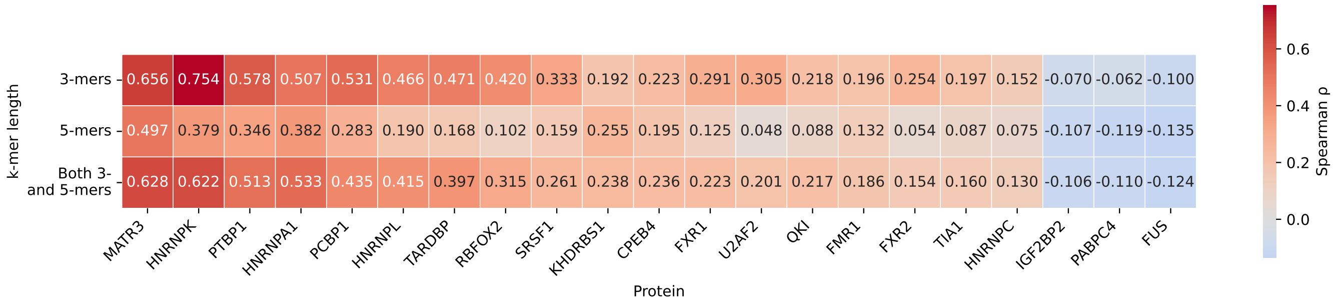

B

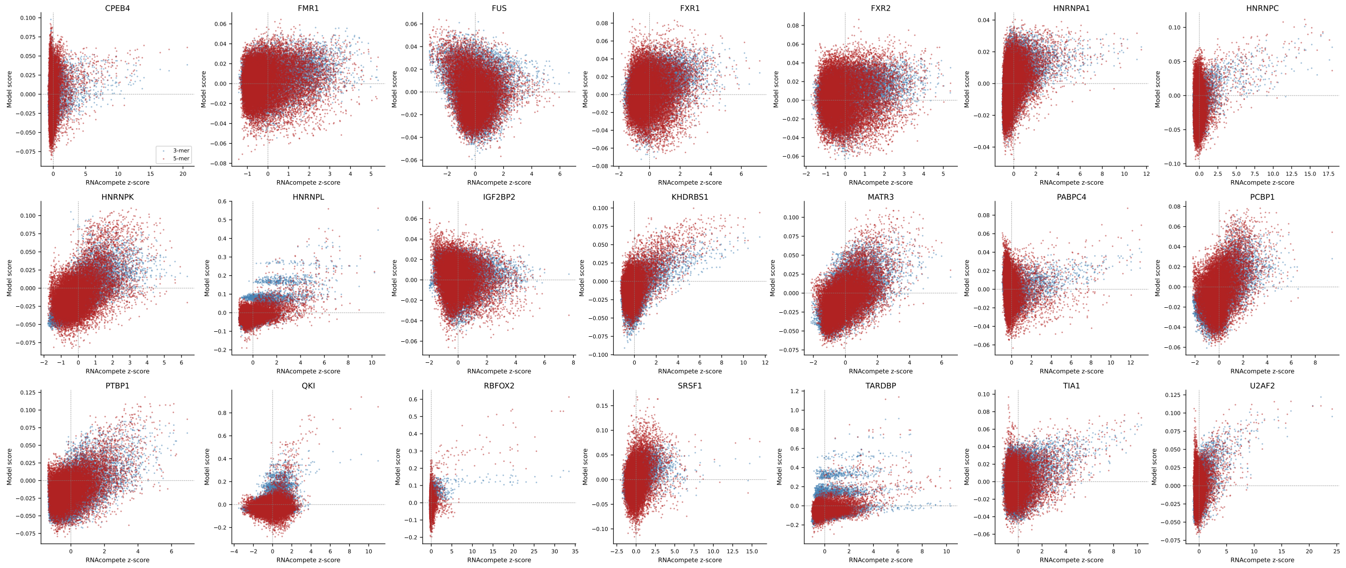

Supplementary Figure S8. Correlation analysis of RNAcompete-based 7-mer z-scores and PLERIO-inferred model scores obtained from 3- and 5-mers. **A** correlation for each of the proteins studied that have both RNAcompete and eCLIP assay data on K562 cell line, **B** scatterplots for RNAcompete 7-mers z-score and PLERIO-inferred eCLIP-based 7-mer score.

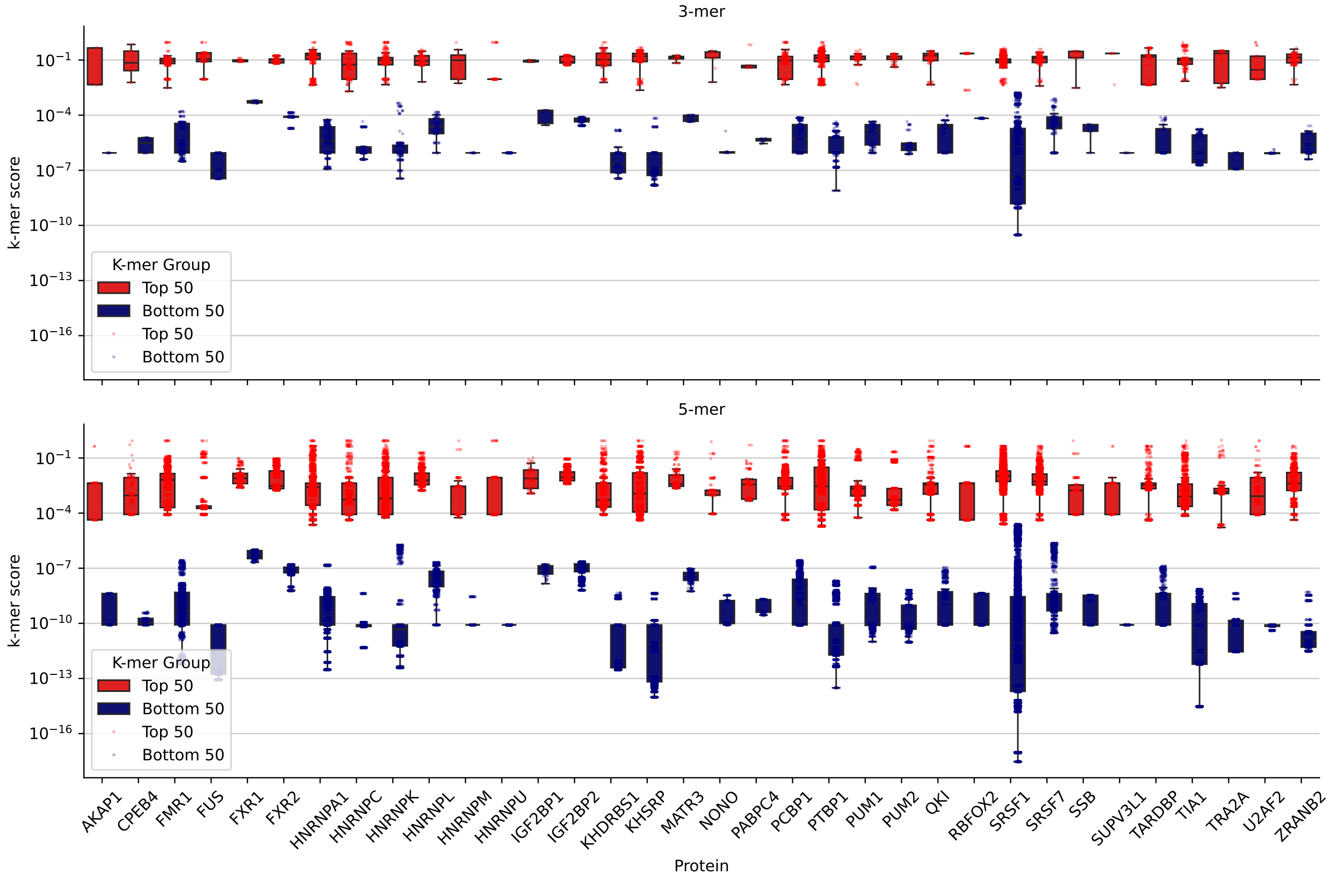

Supplementary Figure S9. PLERIO-inferred k-mers scored by each of the proteins PWMs. Top and bottom 3- and 5-mers from each protein models were scored against a corresponding set of PWMs obtained from ATtRACT database.

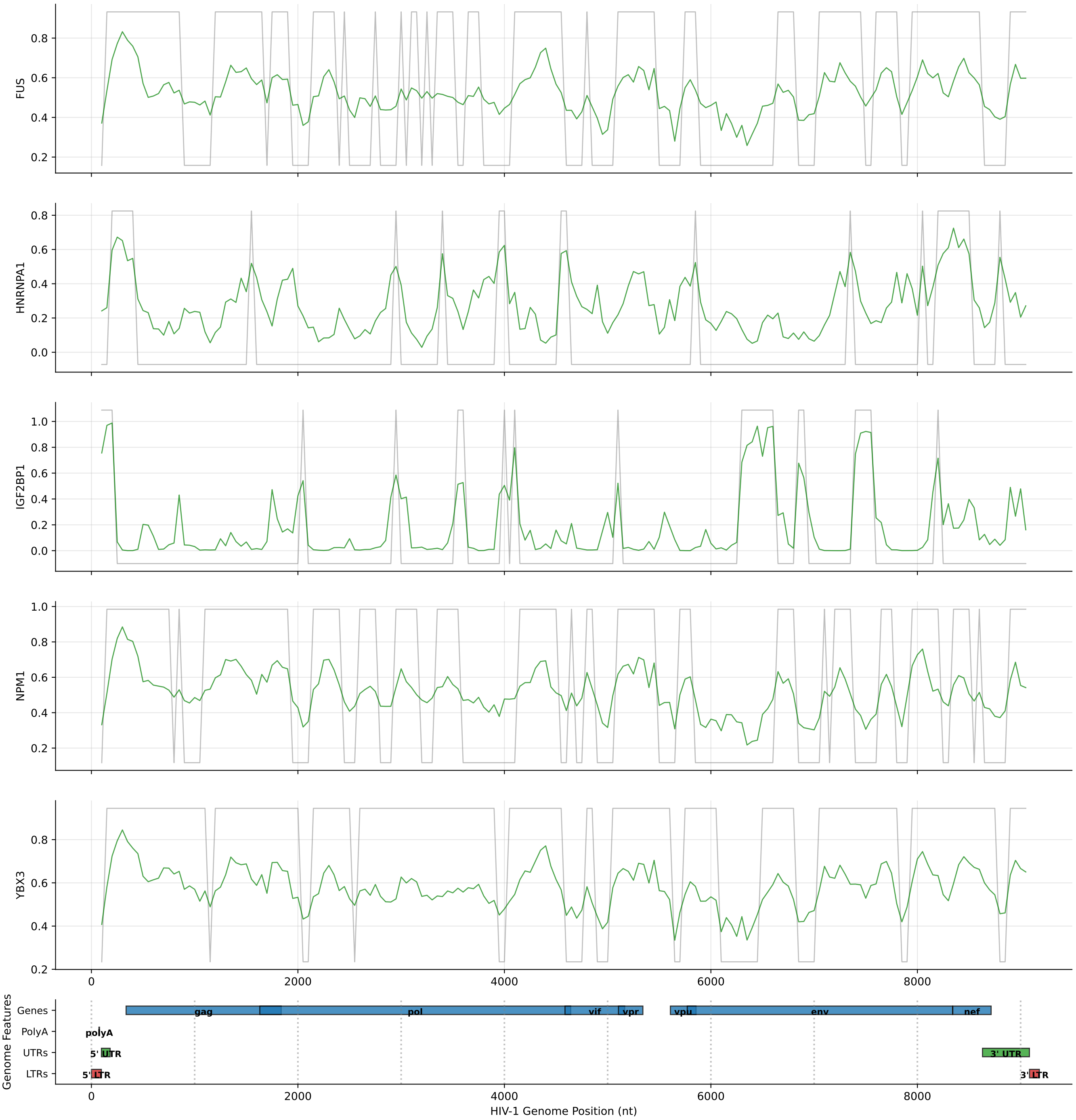

Supplementary Figure S10. PLERIO-inferred probabilities of select proteins to bind HIV-1 RNA. The green track shows probability for sliding window of size 200 bp and a step of 50 bp. Gray track represents the quantized probability of interaction and is equal to 1 if the probability is larger that 0.5.

A

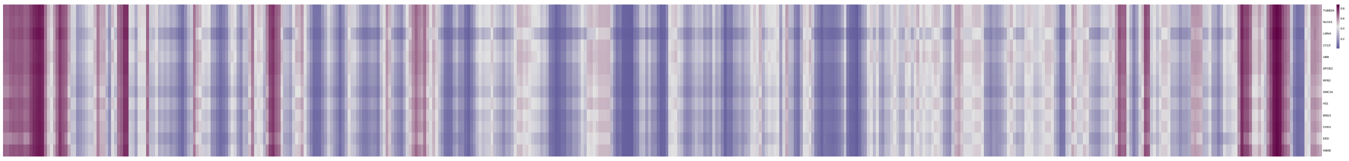

B

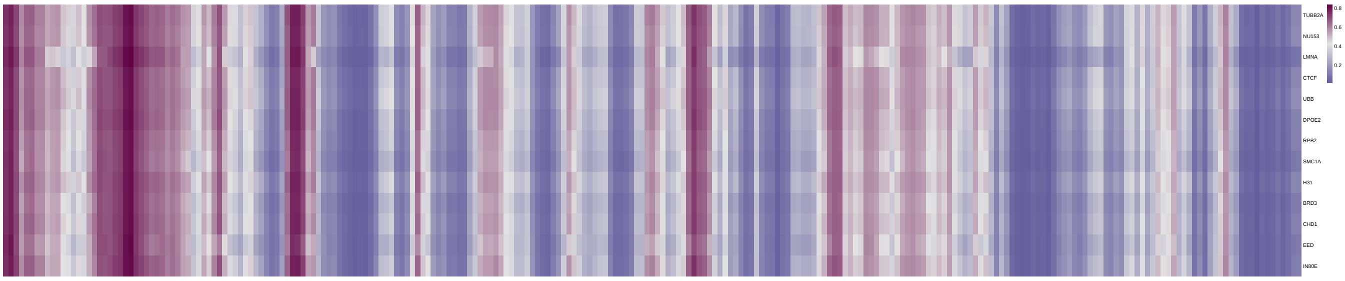

C

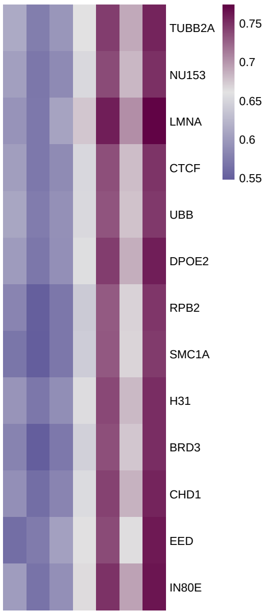

Supplementary Figure S11. Examples of multi-protein model ncRNA-protein contact inference in PLERIO framework: **A** NEAT1; **B** HOTAIR; **C** TERC; with multiple proteins covering a wide range of functions where RNA-binding might not be needed. Yet, the interaction profile is practically the same.

A

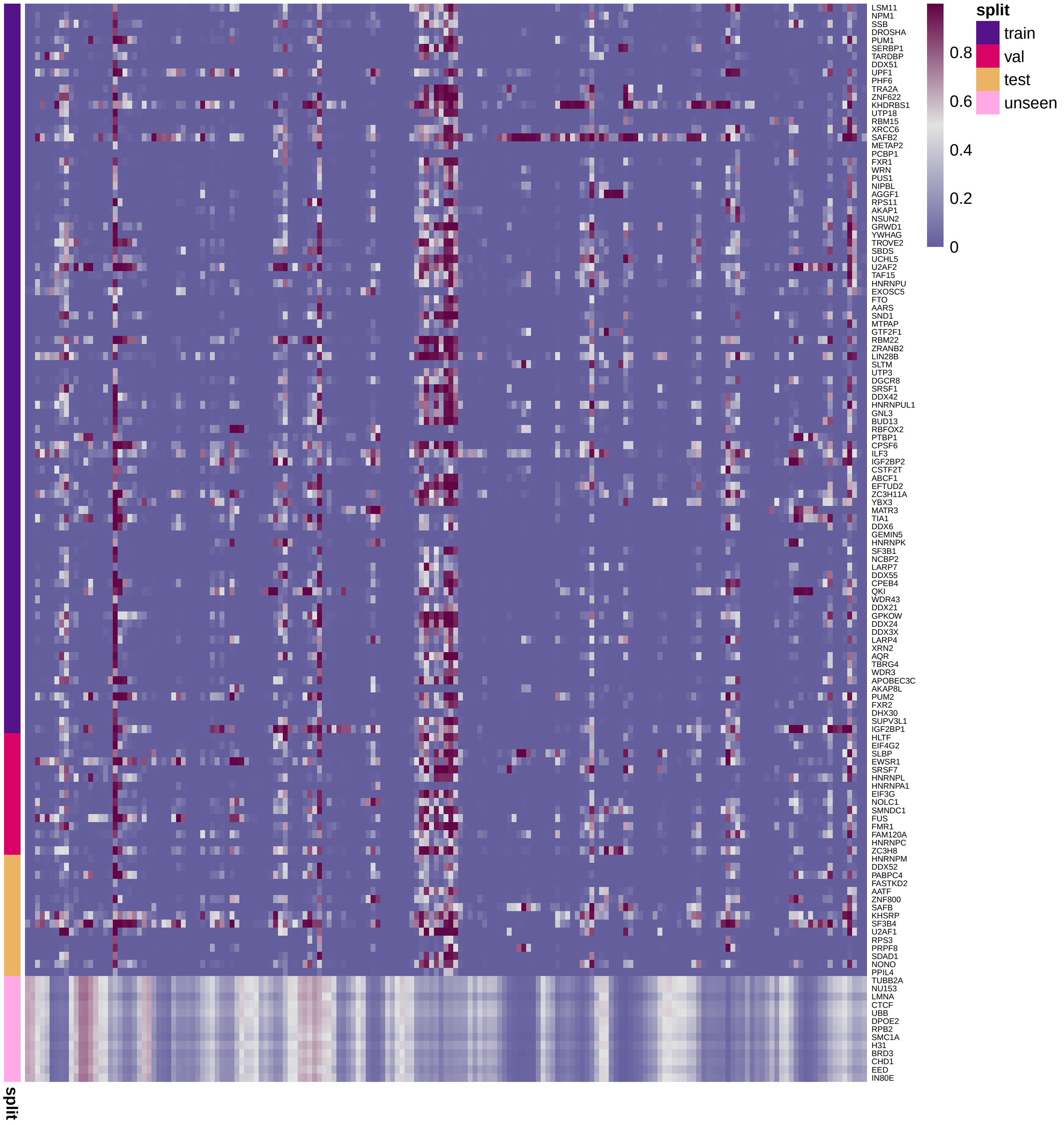

Supplementary Figure S12. Examples of multi-protein model prediction for MALAT1 RNA stratified by a sample of protein: proteins are either from training, testing or independent sets.

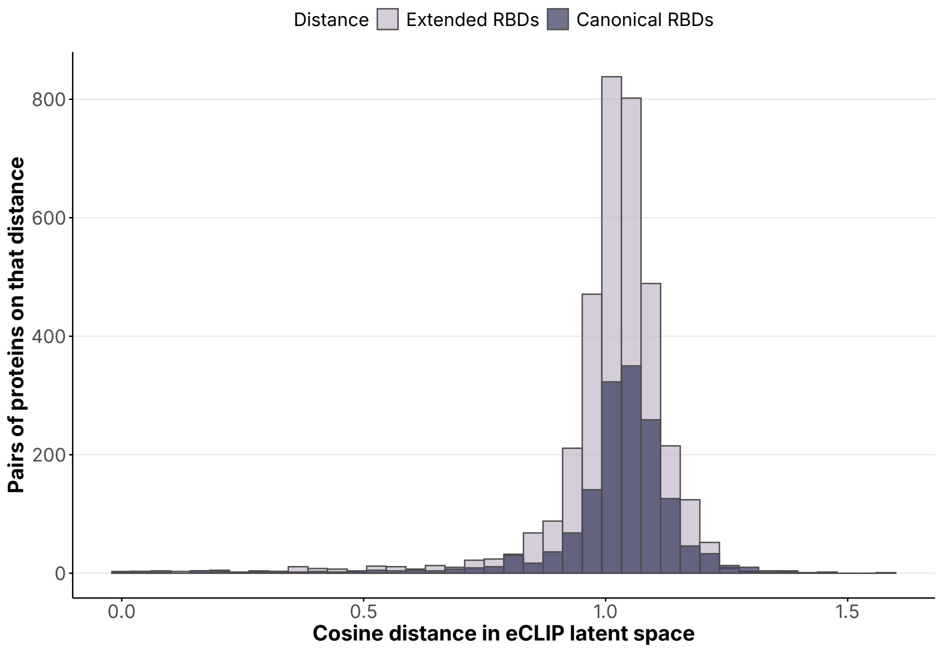

Supplementary Figure S13. Distribution of cosine distances in eCLIP-based latent space obtained by JPLE algorithm on available proteins with a canonical RBD or a domain that can be less strictly considered RNA-binding. Wasserstein's W1 distance between two distributions is 0.01697.
